# FOS3D: A Fluorescence‐Enabled Toolkit for Characterizing a Three‐dimensional Osteosarcoma Model

**DOI:** 10.1002/advs.76031

**Published:** 2026-06-11

**Authors:** William Humble, Wiktor Zywicki, Enrico Lucarelli, Ania Naila Guerrieri, Francesca Taraballi, Gianluca Cidonio, Claudia Di Bella, Carmine Onofrillo, Andrea J. O'Connor, Serena Duchi

**Affiliations:** ^1^ Aikenhead Centre for Medical Discovery (ACMD) St Vincent's Hospital Melbourne Fitzroy Victoria Australia; ^2^ Department of Surgery The University of Melbourne St Vincent's Hospital Melbourne Fitzroy Victoria Australia; ^3^ Department of Biomedical Engineering The Graeme Clark Institute The University of Melbourne Parkville Victoria Australia; ^4^ Osteoncology, Bone and Soft Tissue Sarcomas and Innovative Therapies Unit IRCCS Istituto Ortopedico Rizzoli Bologna Italy; ^5^ Center For Musculoskeletal Regeneration Houston Methodist Research Institute Houston Texas USA; ^6^ Orthopaedics and Sports Medicine Houston Methodist Hospital Houston Texas USA; ^7^ Department of Mechanical and Aerospace Engineering University of Rome La Sapienza Rome Italy; ^8^ Center For Life Nano‐ & Neuro‐ Science‐CLN2S Italian Institute of Technology (IIT) Rome Italy; ^9^ Department of Orthopaedics Sarcoma Unit St Vincent's Hospital Melbourne Fitzroy Victoria Australia

**Keywords:** chemotherapeutics, fluorescence, GelMA, in vitro models, osteosarcoma, tissue engineering

## Abstract

Osteosarcoma (OS) remains a rare, aggressive primary bone malignancy for which therapeutic progress has been limited for several decades. Three‐dimensional (3D) tissue‐engineered models can better reproduce the tumor microenvironment than monolayer cultures; however, their routine use is often constrained by low‐throughput workflows and reliance on destructive analytical endpoints. Here, we present FOS3D, a fluorescence‐enabled OS model based on gelatin methacryloyl hydrogels incorporating stable green fluorescent protein (GFP)‐expressing tumor cells. By varying polymer concentration and photo‐crosslinking duration, hydrogels spanning compressive moduli of approximately 5–50 kPa were generated, covering stiffness ranges reported for OS stromal environments. Whole‐well fluorescence scanning enabled rapid, non‐destructive quantification of proliferation in intact constructs and showed correlation with DNA and metabolic assays. Integration with light‐sheet microscopy further enabled volumetric imaging of tumor organization, revealing time‐dependent changes in spatial distribution. Transcriptional profiling and immunohistochemistry confirmed microenvironment‐driven adaptations associated with extracellular matrix remodeling, stemness, and drug‐resistance pathways. Finally, GFP‐based longitudinal monitoring enabled chemotherapeutic screening of cisplatin and doxorubicin across two OS cell lines, capturing dose‐dependent responses and the increased treatment tolerance characteristic of 3D cultures. Collectively, FOS3D provides a scalable fluorescence‐enabled toolkit for high‐content characterization and drug‐response profiling of OS in physiologically relevant 3D environments.

## Graphical abstract

FOS3D describes fluorescent (F) osteosarcoma (OS) cells in a tri‐dimensional (3D) model. The study comprises three phases: development, where biofabrication parameters are tuned to achieve cytocompatibility and tumor‐specific mechanical properties in cell‐laden gelatin methacryloyl constructs; validation, where whole‐well fluorescence reading is correlated with conventional endpoint assays, metabolic activity, and DNA quantification; and assessment, where light‐sheet microscopy, RT‐qPCR, immunohistochemistry, and drug testing provide high‐content characterization of cell behavior and chemotherapeutic response in the 3D model. Created in BioRender. Research, R. (2026) https://BioRender.com/g0hvnxf.

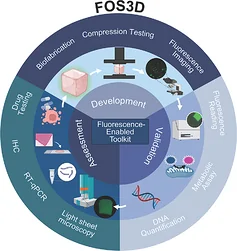

## 1 Introduction

Tissue engineering has enabled the understanding of diseases such as cancer from a more physiologically relevant perspective by incorporating features typical of the 3D in vivo microenvironment. This includes extracellular matrix (ECM) composition, architecture, and mechanical cues that regulate cell behavior [1]. In cancer, the ECM plays central roles in tumor progression by influencing proliferation, invasion, mechanotransduction, and therapeutic response [2]. Hydrogels are widely used to model ECM because they are biocompatible, hydrated, biodegradable, and mechanically tunable [3]. For rare and complex diseases such as OS, in vitro 3D tissue‐engineered models are particularly attractive because they can recreate microenvironmental constraints that are absent in conventional monolayer systems, yet they eschew the complexity and resource intensiveness of animal models. Although animal models provide a 3D and systemic context, they bring important limitations, including interspecies differences in bone biology, tumor evolution, and therapeutic response, as well as ethical considerations, high cost, and limited experimental controllability, which can hinder mechanistic investigation and clinical translation [4]. Thus, in vitro 3D tissue‐engineered models can be used to iteratively build complexity and examine specific disease mechanics in a controlled manner. This is important for enhancing translation and the specificity of drug development, especially considering only 7% of oncological drugs enter clinical trials and receive regulatory approval. The 5‐year event‐free survival rate has remained between 60% and 70% in non‐metastatic OS, and around 25% for metastatic cases, for over 4 decades despite considerable research [5, 6, 7]. Developing alternative drugs is also vital in meeting the challenge of chemoresistance, which is reported in up to 77% of OS cases under current regimens [8]. Clearly, new treatments are needed, and tissue engineering presents a new frontier for OS modelling with the potential to unlock these treatments.

OS is the most common primary bone tumor affecting adolescents and those over 60 [9]. OS originates within the bone from cells of mesenchymal lineage, typically osteoblastic, although it can exhibit pleiotropic phenotypes [10]. Common genetic alterations include disruption of tumor suppressor pathways involving TP53 and RB1, while the production of aberrant osteoid matrix is a defining histological hallmark [11, 12]. OS frequently arises near the metaphyseal growth plate, a region surrounding mineralized bone and trabecular stromal compartments [10]. This stromal environment contains osteoblasts and osteoclasts, immune populations, and extensive vasculature, supporting tumor–stroma interactions that influence bone remodeling, inflammation, immune evasion, and nutrient access [6]. OS is highly prone to hematogenous dissemination, and pulmonary metastasis remains the leading cause of disease‐related mortality [13, 14]. When studying OS, cells can be patient‐derived, but are typically commercially obtained for in vitro studies, with many cell lines such as MG‐63 and Saos‐2, well characterized in the literature and representing unique phenotypic and genetic differences [15].

The ECM of bone stroma is primarily composed of collagen, inorganic minerals (mainly hydroxyapatite), water, and lipids [6, 16]. Hydrogel systems recapitulate aspects of this hydrated matrix and are commonly used in modelling bone marrow and stromal niches [17, 18, 19]. Gelatin methacryloyl (GelMA) is a widely used hydrogel derived from denatured collagen that is methacrylated to enable photo‐crosslinking and is seeing increasing use in 3D in vitro tumor models [20]. In the presence of a photo‐initiator and light exposure, GelMA forms a thermostable, hydrophilic polymer network [21]. Critically, its viscosity, stiffness, and viscoelastic behavior can be modulated by polymer concentration and photo‐crosslinking parameters, enabling tailored environments to examine tumor–matrix interactions [22]. Importantly, hydrogels such as GelMA can recapitulate key features of the OS stromal niche, which represents the soft, organic phase of the bone ECM distinct from the mineralized component. This stromal phase is primarily composed of collagen‐rich organic matrix and proteoglycans and exhibits relatively low stiffness, with reported Young's modulus values in the range of approximately 0.25–24.7 kPa in bone marrow and surrounding stromal tissues, although higher stiffness ranges can occur in tumor‐associated and fibrotic regions [6, 23]. Replicating these mechanical cues is essential, as matrix stiffness, architecture, and biochemical composition are known to regulate tumor cell proliferation, mechanotransduction, and therapeutic response. GelMA is particularly well suited for this purpose because its mechanical properties can be precisely tuned across physiologically relevant ranges, including 5–50 kPa, enabling modelling of stromal‐like and tumor‐remodeled environments while maintaining the collagen‐derived biochemical signals, such as arginine‐glycine‐aspartic acid (RGD) sequences, necessary for cell adhesion and function [21]. This tunability, combined with its biocompatibility and structural similarity to collagen, supports its use as a biomimetic platform for engineering physiologically relevant 3D OS models that capture critical tumor–stroma interactions.

However, a major limitation in the routine adoption of 3D OS models in general is that increasing dimensionality complicates analysis [24]. Imaging resolution and orientation are reduced in thick constructs, while many standard endpoint assays require adaptation to achieve adequate reagent diffusion, lysis efficiency, or biomaterial removal [24, 25]. Such workflows are often destructive and labor‐intensive, limiting throughput and making longitudinal designs challenging. A key bottleneck for 3D OS models is therefore measurement scalability. Accordingly, there is a need for non‐destructive, repeatable, and quantitative readouts that can be acquired rapidly in multi‐well formats while preserving samples for downstream imaging and molecular analysis.

To provide a more convenient method of analysis, fluorescence may be used. Historically, fluorescently labelled cells have been used in animal studies to monitor cell migration and metastasis from xenografted OS tumors [26]. However, fluorescence‐based methods also offer the opportunity to monitor and quantify cellular activity within 3D in vitro models, providing continuous monitoring of cell behavior [27].

Typically, cells can be fluorescently labeled using cell‐permeable dyes or stable genetic approaches. While cytoplasmic dyes enable short‐term tracking, fluorescence intensity dilutes with each division, limiting their use for longitudinal proliferation monitoring [27, 28]. In contrast, viral transduction enables stable expression of fluorescent proteins, maintaining or increasing fluorescence through proliferation and allowing sterile, rapid quantification without disrupting constructs [29, 30]. This fluorescence‐based approach allows continuous, longitudinal monitoring as well as a visual tool without additional staining or processing steps. Fluorescence‐based analysis has more recently been integrated into advanced tumor culture formats such as microfluidic systems [26, 31, 32]. However, the use of fluorescent protein expression as a quantitative proliferation readout in 3D OS models remains underexplored and requires validation against standardized biological assays to establish its use. Fluorescence tracking is especially valuable for 3D drug testing because it enables time‐resolved measurement of response kinetics (onset, lag, rebound) rather than reliance on single endpoints. In OS, matrix confinement, altered mechanosensing, and diffusion barriers can shift drug sensitivity relative to 2D cultures. However, appropriate optical controls are essential because some compounds, such as the chemotherapeutic drug doxorubicin (DOX), exhibit intrinsic fluorescence or can quench fluorescence signals depending on their environment and interactions, potentially confounding intensity‐based readouts and requiring careful spectral and concentration calibration. Orthogonal benchmarking using metabolic and DNA measurements, together with imaging‐based confirmation, strengthens confidence that fluorescence changes reflect biological cytotoxicity/cryostasis rather than optical artefact. Several commonly used fluorescent proteins exist; however, green fluorescent protein (GFP) is particularly attractive due to its stability, strong signal, and widespread compatibility with imaging platforms [33]. GFP‐labelled OS cells have been used in animal xenograft models for growth and metastasis tracking, yet there is limited demonstration of GFP as a non‐destructive proliferation metric specifically in 3D OS hydrogels. Addressing this gap would provide a convenient, scalable readout compatible with both high‐throughput multi‐well scanning and high‐content fluorescence imaging such as light‐sheet microscopy. This approach is inherently scalable because large numbers of samples and experimental conditions can be analyzed in parallel using standard multi‐well plate formats, enabling higher‐throughput and more cost‐effective studies than animal models, and making it particularly well suited for applications such as drug screening and combinatorial testing.

Here, we present FOS3D—Fluorescent Osteosarcoma 3D model, a mechanically tunable GelMA‐based OS model integrating stable GFP expression with two complementary fluorescence modalities: (i) high‐throughput, longitudinal proliferation kinetics in multi‐well format, and (ii) whole‐construct volumetric fluorescence imaging (light‐sheet microscopy) to resolve spatial distribution, clustering, and morphology of cells in 3D over time. We validated GFP intensity as a proxy for cell number in both 2D and 3D by benchmarking against DNA content and metabolic activity assays. We then applied the platform to characterize time‐dependent tumor organization, and we coupled phenotypic fluorescence tracking with RT‐qPCR profiling to confirm transcriptional adaptations induced by 3D culture relative to 2D monolayers. We also confirmed through immunohistochemistry (IHC) analyses the expression of ABCB1 (P‐glycoprotein), a well‐established prognostic biomarker for OS [34, 35, 36]. Finally, we quantified chemotherapeutic responses to frontline agents, cisplatin (CDDP) and DOX across two OS cell lines cultivated in our FOS3D and 2D monolayer (MG‐63‐GFP and Saos‐2‐GFP). Collectively, the FOS3D workflow provides a scalable toolkit for mechanistically informed characterization and drug‐response profiling of OS in physiologically relevant 3D environments while preserving samples for downstream analyses.

## 2 Results

### 2.1 Biofabrication of a Mechanically Tunable GelMA‐Based 3D Tissue‐Engineered Osteosarcoma Model

**FIGURE 1:**
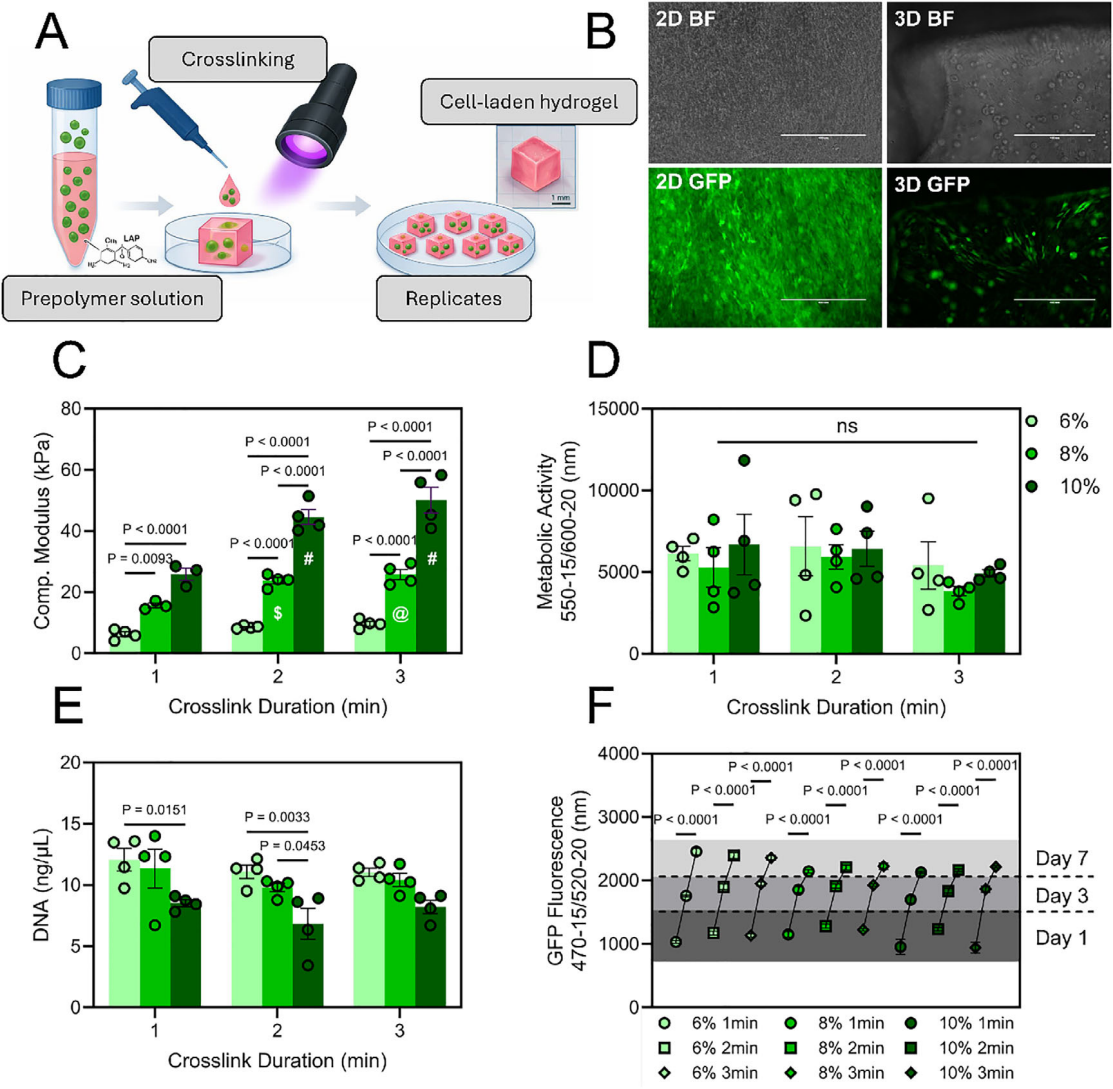
Biofabrication and characterization of a tunable 3D GelMA‐based osteosarcoma model (FOS3D). (A) Schematic representation of the biofabrication workflow. MG‐63‐GFP tumor cells are suspended in a GelMA–LAP prepolymer solution, mixed to form a homogenous cell‐laden precursor, and 35 µL is dispensed into 3.3 mm^3^ PDMS molds. Controlled 405 nm light exposure at 9–10 mW/cm^2^ using LunaCrosslinker (Gelomics, Australia) initiates rapid photo‐crosslinking, forming stable cell‐laden hydrogels. By varying GelMA concentration and crosslinking duration, constructs with defined mechanical properties are produced and subsequently cultured for longitudinal biological assessment. (B) Day‐7 brightfield (BF) and fluorescence images (GFP) of MG‐63‐GFP cells either plated on a 96‐well plate (2D) or encapsulated in 6% GelMA hydrogel (3D). (C) Day‐1 compressive modulus of GelMA hydrogels with varying w/v polymer concentrations (6% light green, 8% green, 10% dark green) and crosslinking durations (1, 2, and 3 min). (D) Day‐7 metabolic activity (fluorescence, 550–15/600–20 nm). (E) Day‐7 DNA content analysis of hydrogels containing MG‐63‐GFP cells (initial cell number 70 000 cells/hydrogel). (F) GFP fluorescence intensity (470–1550/20 nm) measured on day 1, 3, and 7 (separated by shading and dotted lines). Crosslinking durations indicated by symbol shape; 1 min (circle), 2 min (square), 3 min (diamond). Statistical testing involved Two‐way ANOVA & Tukey's Test, *n* = 4 for each condition (GraphPad Prism, USA). $ = *p *> 0.05, @ = *p *> 0.001, # = *p* > 0.0001 versus 1 min crosslinking duration. a.u. = arbitrary units. GFP = green fluorescent protein. ns = non‐significant. Scale bar: (B) 400 µm.

**FIGURE 2:**
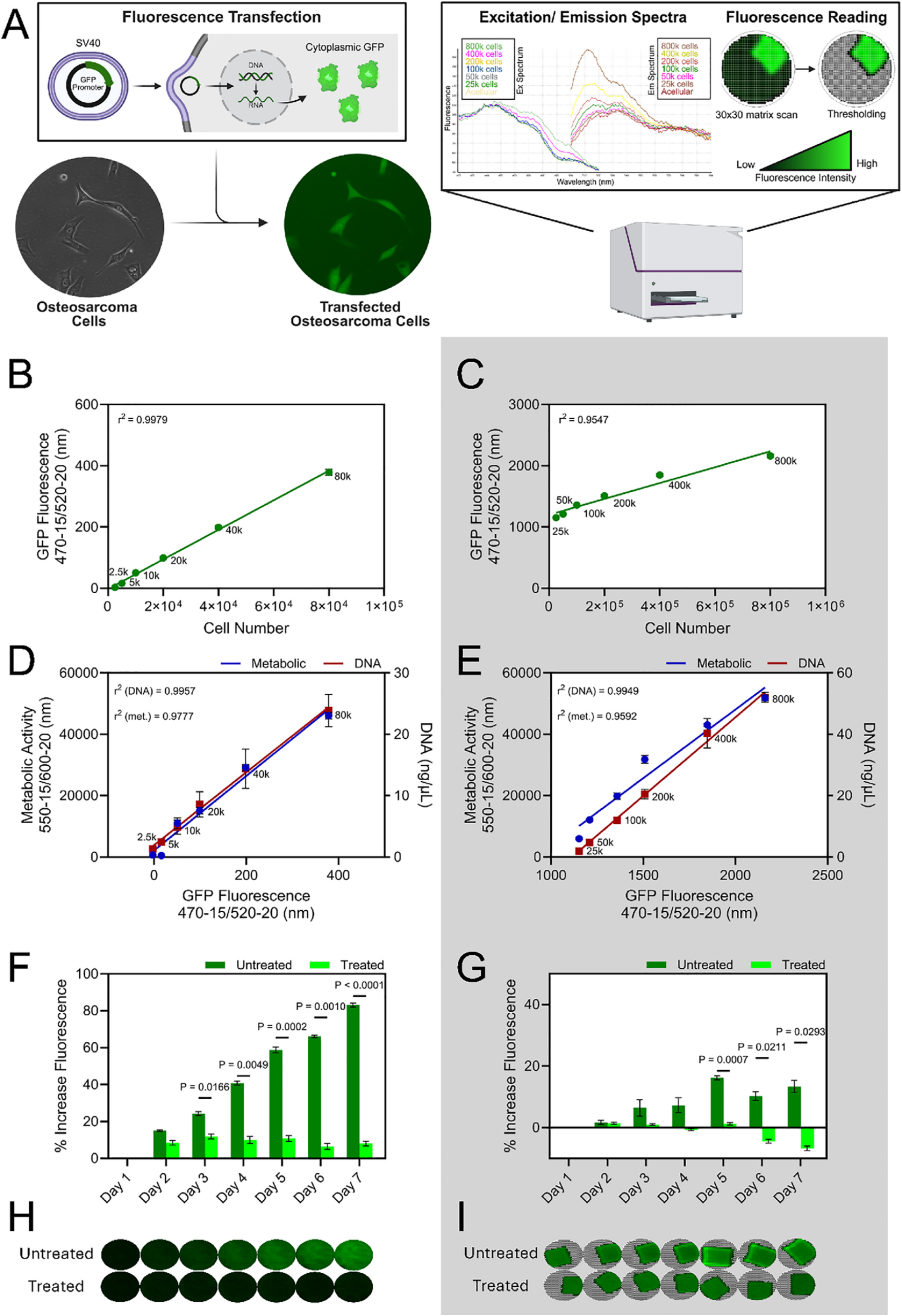
Validation of GFP as a Real‐Time Three‐dimensional Assessment of Proliferation in 2D and FOS3D. (A) OS cells were stably transfected with a GFP reporter gene under the control of the highly active simian virus 40 (SV40) promoter, enabling constitutive cytoplasmic GFP expression across the cell population. Fluorescence intensity is measurable using a plate reader across the appropriate excitation/emission peak (470‐15/520‐20 nm), with each of the colored lines on the fluorescent spectra equating to increasing cell numbers, with a higher peak equating to greater number and therefore fluorescence intensity (BMG Labtech, Germany). In 3D studies, a matrix of 30 × 30 map scan generates a topographical measurement of each cell‐laden hydrogel within a 96‐well plate at a given focal height (4 mm). The scan can be processed to remove background by setting an intensity threshold (1000 a.u.), giving an average fluorescent intensity for the cells within the hydrogel. GFP fluorescence intensity readings of increasing cell number in 2D (B) and 3D hydrogels (C) demonstrate positive linearity. (D) Linear regression analyses of Cell TiterBlue metabolic assay (Promega, USA) and DNA quantification (Invitrogen, USA) against GFP fluorescence in both (D) 2D and (E) 3D. (F) Seven‐day quantitative fluorescence measurement showing increasing GFP fluorescence in untreated samples and samples treated with cytostatic drug (Paclitaxel) compared to day 1 in both 2D and (G) 3D conditions. Representative well scans mapping GFP fluorescence intensity for (H) 2D and (I) 3D conditions. In (B‐ E), simple linear regression analysis and Pearson's correlation are reported as *r* ^2^, *n* = 3. In (F,G), *P*‐values were obtained from two‐way ANOVA along with Šídák's multiple comparisons test, and percentage increase in fluorescence is based on day 1 Untreated values (Graphpad Prism, USA). a.u. = arbitrary units. GFP = green fluorescent protein. ns = non‐significant. Created in BioRender. Research, R. (2026) https://BioRender.com/pa0ghxm.

To develop a mechanically tunable 3D tissue‐engineered model of OS (FOS3D), we employed the MG‐63‐GFP human OS cell line and embedded these cells within GelMA hydrogels biofabricated through a standard mold casting with photo‐crosslinking process (Figure 1A). MG‐63 cells were transfected with GFP, as detailed in the Experimental Section (Figure 2A), to enable real‐time visualization of cellular distribution and morphology throughout the culture period [37, 38]. The resulting MG‐63‐GFP cells were homogeneously suspended in a GelMA–LAP prepolymer solution, and 35 µL was dispensed into PDMS molds of 3.3 mm^3^ cubic constructs and photo‐crosslinked under precisely timed 405 nm light exposure. By adjusting GelMA concentration (6%, 8%, and 10%) and crosslinking duration (1–3 min), we generated a library of constructs with distinct mechanical profiles while maintaining consistent cell encapsulation. As shown in Figure 1B, GFP labelling highlights clear differences in cell morphology and spatial organization between 2D monolayer culture and encapsulation within the 3D GelMA hydrogels. Importantly, these comparisons were performed under matched theoretical cell–cell spacing between formats (as described in the Experimental Section), where the average center‐to‐center distance between cells was estimated assuming homogeneous distribution across either a 2D plane or a 3D volume (Equations 1 and 2). Under these conditions, by day 7, cells cultured in 2D exhibited a relatively uniform morphology dominated by elongated, spindle‐like spreading, consistent with monolayer growth on a rigid substrate. In contrast, cells within 3D constructs displayed a more heterogeneous, tumor‐like phenotype, with a mixed population of rounded cells and elongated/spreading cells, reflecting matrix confinement, 3D cell–ECM interactions, and spatially regulated growth typical of solid tumor microenvironments (See representative pictures in Figure 1B).

Mechanical characterization confirmed that both polymer concentration and crosslinking duration significantly modulated hydrogel stiffness (Figure 1C). Compressive modulus increased markedly with higher GelMA concentration across all crosslinking durations, with the largest differences observed between 6% and 10% formulations. Extending light exposure duration from 1 to 2 or 3 min further elevated stiffness within the 10% GelMA group, indicating that highly concentrated formulations remain responsive to additional network formation. In contrast, 6% and 8% GelMA reached near‐plateau stiffness earlier, suggesting a more limited crosslinking capacity at lower polymer densities. The compressive moduli ranged from 6.21 ± 1.6 to 50.1 ± 8.3 kPa, as summarized in Table 1. Importantly, these values fall within the stiffness range reported for the bone marrow stromal phase and tumor‐associated microenvironments, confirming that the engineered GelMA constructs replicate relevant mechanical features of the OS niche [6].

**TABLE 1:** Day 1 mean compressive modulus of hydrogels with increasing GelMA concentration and 405 nm crosslinking duration. Note: Mean ± standard deviation, *n* = 4 technical replicates. Statistical analysis provided in Figure 1C.

Crosslinking duration		w/v GelMA (%)	
	6%	8%	10%
1 min	6.21 ± 1.6	18.5 ± 5.7	25.9 ± 3.5
2 min	8.60 ± 0.5	23.8 ± 2	44.6 ± 4.9
3 min	9.69 ± 1.4	25.8 ± 3.3	50.1 ± 8.3

Despite these substantial mechanical differences, tumor cell metabolic activity on day 7 remained statistically comparable among all conditions (Figure 1D). Although variability was noted within groups, no significant effects of GelMA concentration or crosslinking duration were detected, indicating that cell viability is preserved across the full range of tested stiffnesses. DNA quantification revealed a modest but notable sensitivity to polymer concentration, with lower GelMA concentrations (6% and 8%) supporting higher DNA content than 10% hydrogels after shorter crosslinking durations (Figure 1E). These differences diminished in the 3‐minute crosslinking condition, suggesting that extended photo‐crosslinking reduces the impact of matrix density on proliferative capacity. Longitudinal metabolic activity measured as GFP intensity demonstrated a consistent increase in fluorescence from day 1 to days 3 and 7 across all groups (Figure 1F). This uniform rise confirms that encapsulated tumor cells remained metabolically active and proliferative regardless of the mechanical environment. Based on these findings, 6% GelMA crosslinked for 2 min was selected for all subsequent experiments, as it provided the most favorable balance between mechanical robustness for handling/longitudinal testing and a microenvironment permissive to tumor cell expansion (higher DNA content), while maintaining strong GFP‐based proliferation signals.

### 2.2 Validation of GFP as a Real‐Time Three‐dimensional Assessment of Proliferation in 2D and FOS3D

To evaluate whether GFP fluorescence could serve as a reliable indicator of OS cell proliferation in 3D, MG‐63‐GFP cells were seeded at different cell numbers, and their proliferative behavior was assessed (Figure 2A). Importantly, the proliferation rate of GFP‐labelled cells relative to their unlabeled parental counterparts has previously been shown to be comparable, as reported by Lenna et al. [39]. Panel A illustrates how plate‐reader measurement of GFP expression generates a quantifiable fluorescence spectrum within the excitation/emission peak of 470–15/520–20 nm, with emission peak height increasing proportionally with cell number. During acquisition, a 30 × 30 scan matrix is generated for each well, providing both quantitative numerical data and a qualitative topographical readout of signal intensity. In 2D cultures, GFP signal scaled linearly with cell number (*r* ^2^ = 0.9979, *p* < 0.0001), demonstrating a strong relationship between fluorescent output and cell number (Figure 2B). Notably, 3D GelMA cultures also exhibited a strong linear correlation between GFP intensity and cell (*r* ^2^ = 0.9547, *p* = 0.0008), indicating reliable fluorescence‐based quantification even within a volumetric matrix (Figure 2C). When GFP fluorescence was compared to conventional proliferation assays, both DNA and metabolic activity content showed excellent agreement with GFP signal in 2D (*r* ^2^ = 0.9957, *p* < 0.0001 and *r* ^2^ = 0.9777, *p* = 0.0002, respectively; Figure 2D). The same trend held in 3D, where DNA content and metabolic activity were strongly correlated with GFP signal (*r* ^2^ = 0.9949, *p* < 0.0001 and *r* ^2^ = 0.9592, *p* = 0.0006, respectively; Figure 2E). These results validate GFP fluorescence as a robust approach to longitudinal proliferation monitoring in both 2D and 3D systems.

Longitudinal monitoring over 7 days revealed marked differences in proliferative behavior between 2D and 3D environments, as demonstrated by both quantitative fluorescence measurements and spatial fluorescence mapping imaging (Figure 2F–I). In untreated conditions, MG‐63‐GFP cells cultured in 2D exhibited a rapid and sustained increase in GFP fluorescence, reaching an 83.1% increase by day 7 (*p* < 0.0001), consistent with monolayer expansion (Figure 2F,H). In contrast, untreated cells embedded within 3D GelMA hydrogels displayed a significantly attenuated proliferative profile, with only a 13.4% increase in fluorescence by day 7 and a peak at day 5 (16.2%, *p* < 0.01) (Figure 2G,I). Spatial fluorescence mapping confirmed these findings, showing homogeneous surface expansion in 2D cultures, whereas in 3D constructs, GFP‐positive cells were localized at the hydrogel edges from day 4, suggesting preferential growth in regions with greater nutrient and oxygen accessibility, a feature commonly observed in tumors (Figure 2I) [40]. These findings also indicate that the 3D hydrogel matrix imposes biomechanical and spatial constraints imposed by the hydrogel microenvironment, whose stiffness falls within the physiologically relevant range of the stromal phase of bone marrow [23]. To further contextualize this restrained growth, the temporal evolution of hydrogel mechanical properties was evaluated (Figure S1). Compressive modulus measurements demonstrated a reduction in matrix stiffness over time, with significantly higher stiffness at day 1 compared to later timepoints (*p* < 0.001), confirming that cells were initially cultured within a mechanically restrictive environment that gradually softened during culture, but remain stable over 21 days and within the range of the OS stromal phase.

Following paclitaxel treatment, GFP fluorescence increases were significantly suppressed in both systems, confirming its cytostatic effect. In 2D cultures, paclitaxel reduced fluorescence to minimal levels, with treated samples showing only a 6.5% increase by day 7 compared to 83.1% in untreated controls (*p* < 0.0001) (Figure 2F,H). In 3D constructs, paclitaxel similarly inhibited proliferation, with fluorescence remaining near baseline or decreasing slightly over time, resulting in significantly lower values than untreated controls from day 5 onward (Figure 2G,I). Notably, the overall proliferative activity and treatment response remained lower in 3D than in 2D, highlighting the moderating influence of the 3D matrix.

Together, these findings demonstrate that GFP fluorescence enables sensitive, non‐destructive monitoring of OS proliferation and therapeutic response while confirming the critical role of the biomimetic mechanical microenvironment in regulating tumor growth dynamics.

### 2.3 Visualizing and Quantifying Cells in FOS3D Using Light‐Sheet Microscopy

To validate that GFP fluorescence could be used not only to monitor proliferation but also to quantify cell number in 3D, we employed GFP reading and light‐sheet fluorescence microscopy in combination with digital volumetric reconstruction. This approach enabled high‐resolution visualization of MG‐63‐GFP cells throughout the entirety of the GelMA hydrogel constructs without physically sectioning the samples.

**FIGURE 3:**
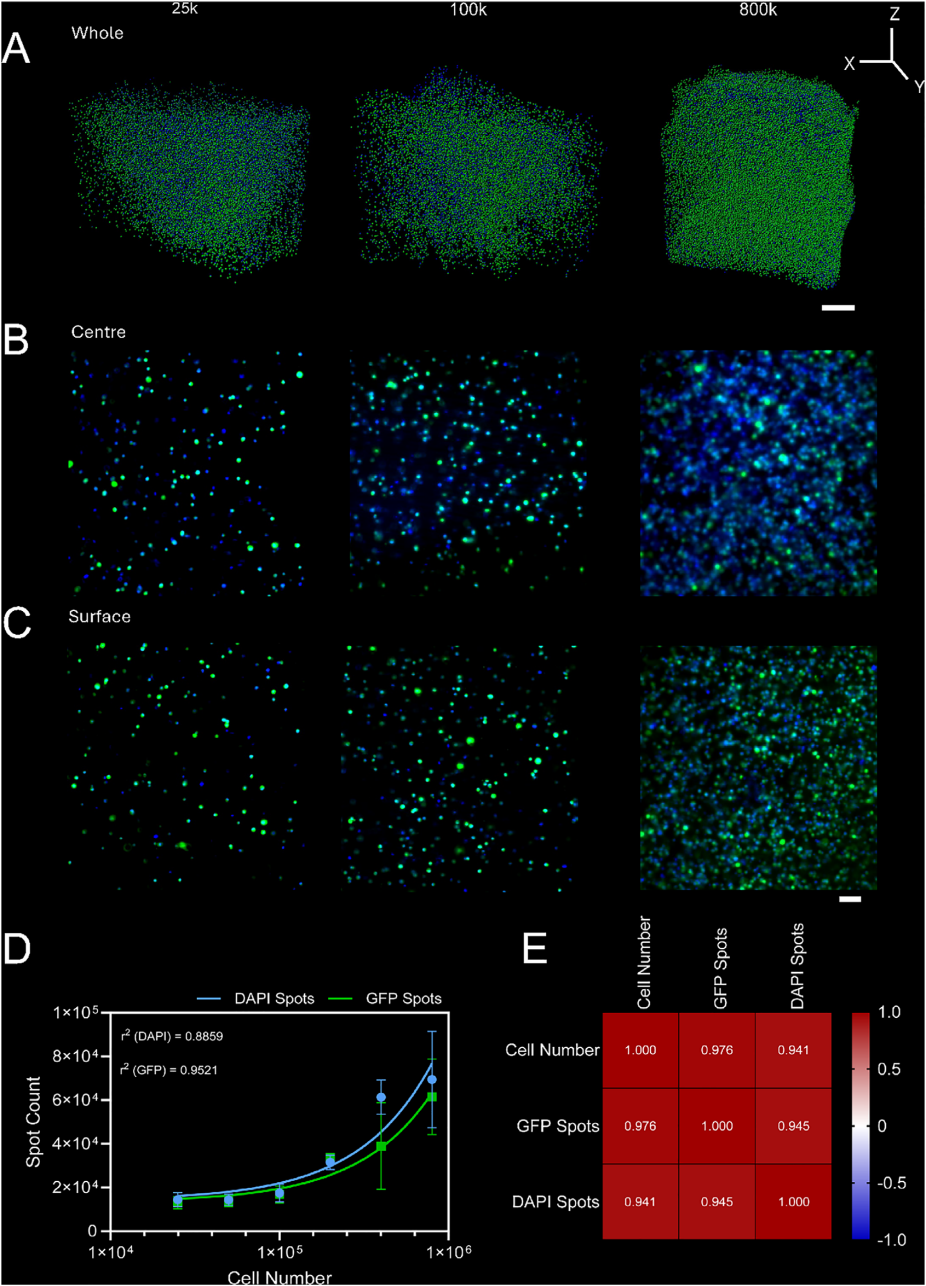
Visualizing and quantifying cell number in FOS3D using light‐sheet microscopy. (A) Day 1 light‐sheet fluorescence microscopy of GelMA hydrogels encapsulating increasing numbers of fluorescence expressing MG‐63‐GFP cell number (25k, 100k, and 800k). The image depicts cytoplasmic expression of GFP (green) and DAPI‐stained nuclei (blue) signal converted to spots via the inbuilt Spot function of Imaris 10 (Oxford Instruments, UK). Acquisition: 4× magnification, GFP (488 nm), DAPI (405 nm) laser, Z‐step 20 µm (Miltenyi Biotec, Germany). (B) High‐magnification Z‐slices taken from the center (1 mm depth) and (C) surface of cell‐laden GelMA hydrogels depicting cytoplasmic expression of GFP (green) and DAPI‐stained nuclei (blue). Acquisition: 12× magnification, GFP (488 nm), DAPI (405 nm) laser (Miltenyi Biotec, Germany). (D) Linear regression analysis of DAPI (blue) and GFP (green) Spot counts across constructs with progressively increasing cell numbers demonstrating a strong positive relationship with cell number. (E) Correlational matrix shows good agreement between nuclear (DAPI) and GFP‐based quantification, validating GFP fluorescence expression as a reliable, non‐destructive proxy for differentiating cell number between 3D constructs. Statistical analysis involved linear regression analysis with goodness of fit reported as *r* ^2^, and Pearson correlation With *r*‐values reported in each square, *n* = 6 (Graphpad Prism, USA). GFP = green fluorescent protein, DAPI = 4′,6‐diamidino‐2‐phenylindole. Scale bars: (A) 500 µm, (B,C) 150 µm.

Plate reading and light‐sheet imaging revealed clear differences in cell number and localization across hydrogels loaded with increasing cell numbers (25k, 50k, 100k, 200k, 400k, and 800k; Figure 3D). Digital reconstruction using Imaris 10 (Oxford Instruments, UK) allowed segmentation and volumetric rendering of GFP‐expressing cells, providing a direct visual representation of how cell packing increased with higher initial seeding (Figure 3A). At lower cell number (25k), cells were sparsely and evenly dispersed, whereas at higher numbers (800k) cells exhibited a more compact, tissue‐like arrangement.

To further interrogate spatial distribution, high‐magnification Z‐slices were extracted from the center (Figure 3B) and surface regions (Figure 3C) of each hydrogel. These images revealed that the distribution of GFP‐positive cells remained largely homogeneous across the hydrogel depth, although higher‐cell‐number constructs displayed regions of increased clustering, consistent with the expected behavior of OS cells in confined 3D environments [41, 42]. Quantification of cell number from 3D reconstructions was achieved by spot detection of DAPI‐stained nuclei and GFP‐positive voxels (Figure 3D). Both DAPI and GFP counts increased proportionally with cell loading, while not necessarily reaching equivalent values. Linear regression analysis demonstrated slightly stronger agreement between GFP spot count and total cell number (*r* ^2^ = 0.9521, *p* = 0.0009) than DAPI spot count and total cell number (*r* ^2^ = 0.8859, *p* = 0.0051). This relationship was further confirmed by correlation analysis, which showed a very strong positive association between total cell number, GFP spots (*r* = 0.976, *p* = 0.001), and DAPI spots (*r* = 0.941, *p* = 0.005), as well as between GFP and DAPI counts themselves (*r *= 0.945, *p *= 0.004) (Figure 3E). These findings confirm that GFP signal accurately reflects total cell number in 3D constructs and can serve as a reliable, non‐destructive proxy for cell quantification.

### 2.4 Longitudinal Visualization of Cell Distribution in FOS3D Using Light‐Sheet Microscopy

**FIGURE 4:**
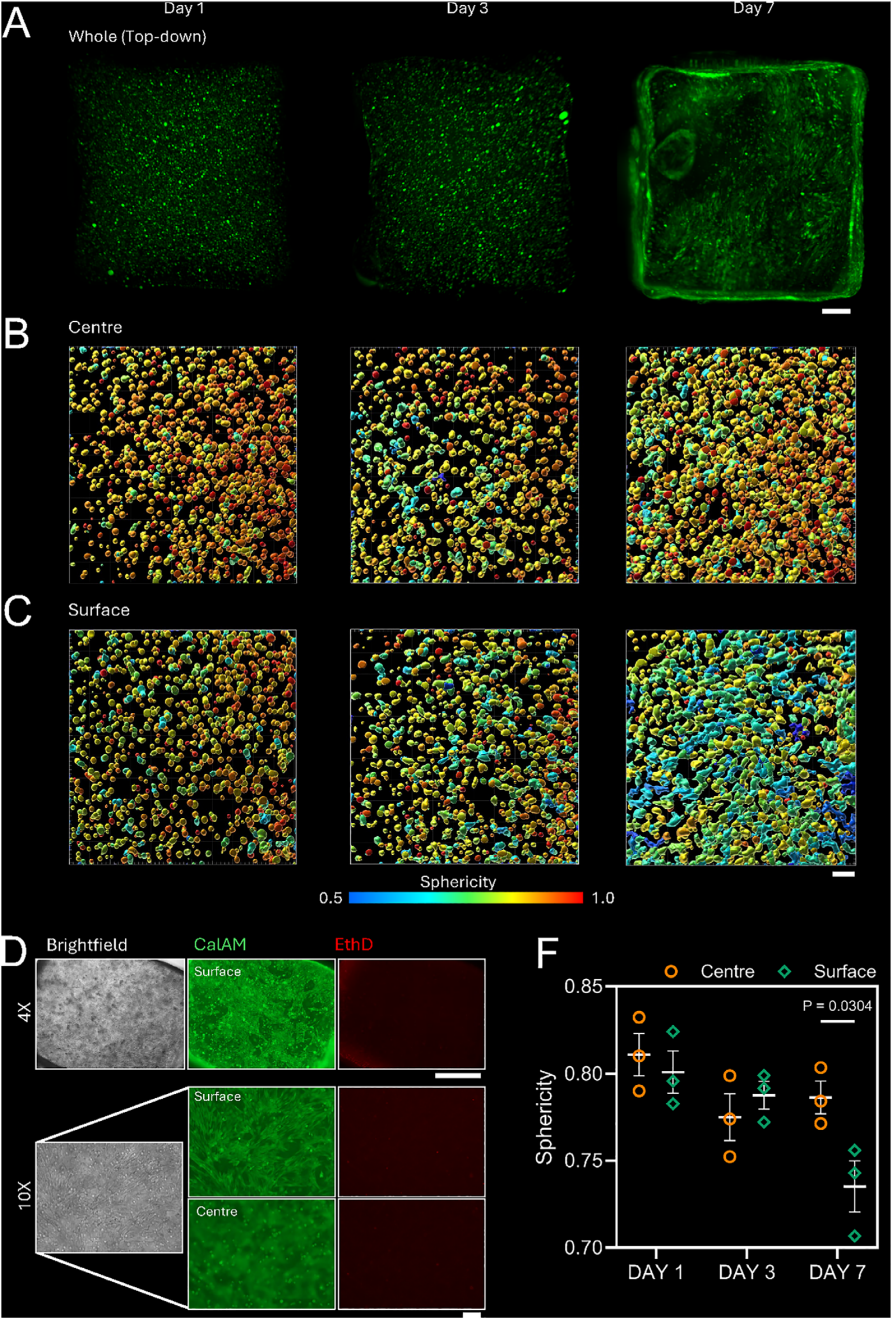
Longitudinal visualization of cell distribution in FOS3D using light‐sheet microscopy. (A) Top‐Down view of light‐sheet microscope images capturing fluorescently transfected MG‐63‐GFP cells in 35 µL GelMA hydrogel over seven days culture. Acquisition: 4× magnification, GFP (488 nm) laser, z‐step 20 µm (Miltenyi Biotec, Germany). (B,C) Centre (1 mm depth) and surface renderings demonstrating cell morphological changes on Days 1, 3, and 7. Renderings are generated using the surface function of Imaris 10 (Oxford Instruments, UK). Color coding indicates the degree of sphericity of the volume object according to Wadell's formula; red = 1 = spherical, blue = 0.5 = spread. Acquisition: 12× magnification, GFP (488 nm) laser, z‐step 20 µm, stack height 500 µm (Miltenyi Biotec, Germany). (D) Live/Dead imaging of MG‐63‐GFP cells (green) in 35 µL GelMA hydrogel after seven days culture. Images captured of the hydrogel surface in 4× images, and the hydrogel surface or ∼100–200 µm below the surface in 10× images. Live cells are stained with calcein acetoxymethyl ester (CalAM; green), while dead cells are stained with ethidium homodimer (EthD; red). Acquistion: 4× and 10× magnification, bright‐field, GFP, and RFP lasers used, respectively (Nikon, Japan). (E) Graph compares average sphericity values of volume objects captured From the center and surface of hydrogels at Days 1, 3, and 7. Multiple *t*‐test, *n* = 3 (Graphpad Prism, USA). Scale bars: (A) 300 µm, (B,C) 500 µm, (D) 500 µm (4×) and 200 µm (10×).

To assess how OS cells reorganize, proliferate, and remodel their 3D environment over time, light‐sheet fluorescence microscopy was employed to image 3DFOS at days 1, 3, and 7. GFP expression enabled 3D longitudinal tracking of cell localization without sectioning, providing a true spatiotemporal view of OS behavior in 3D. Whole‐construct light‐sheet images revealed progressive changes in cellular organization over the culture period. On day 1, cells appeared sparsely and uniformly dispersed throughout the hydrogel, reflecting the initial encapsulation state post‐biofabrication (Figure 4A). By day 3, local increases in GFP signal intensity indicated the onset of proliferation, with emerging micro‐clusters forming at both the central and peripheral regions of the construct. By Day 7, constructs displayed substantially denser fluorescence patterns consistent with continued proliferation and matrix adaptation, showing early signatures of tumor‐like cellular congregation and spreading at the hydrogel periphery (Videos S1 and S2).

To examine temporal changes in cell morphology more closely, higher‐magnification Z‐slices were extracted from the center and surface regions at each time point and reconstructed using the in‐built Surface Statistics function in Imaris 10 (Oxford Instruments, UK; Videos S3 and S4). This function produces color‐coded surfaces based on Wadell's sphericity formula. Less spherical objects are given a value lower than 1 and appear blue, while those closer to a perfect sphere (1) appear red (Figure 4B,C) [43]. Center‐region images showed that MG‐63‐GFP cells gradually increased in number and cluster size from day 1 to day 7, suggesting active proliferation even in regions subjected to reduced nutrient diffusion. Surface‐region images displayed similar trends, though GFP signal intensities increased more at the periphery, reflecting greater nutrient and oxygen availability and consistent with early tumor expansion patterns observed in vivo [44]. Cell sphericity, particularly in the center region, could indicate cellular stress and potential apoptosis. However, Live/Dead imaging revealed a good level of retained viability at the surface‐ and center‐regions, with minimal cell death (Figure 4D). Quantitative analysis of sphericity values demonstrates that center and surface sphericity are most divergent at day 7 (Figure 4F; Figure S2). The difference between center and surface morphology was further assessed with Live/Dead imaging with confocal microscopy which confirmed the observation that cells tend to aggregate deeper within the scaffold, and spread at the periphery, while maintaining good viability in both cases (Figure S3).

### 2.5 Transcriptional and Prognostic Profiling of FOS3D

**FIGURE 5:**
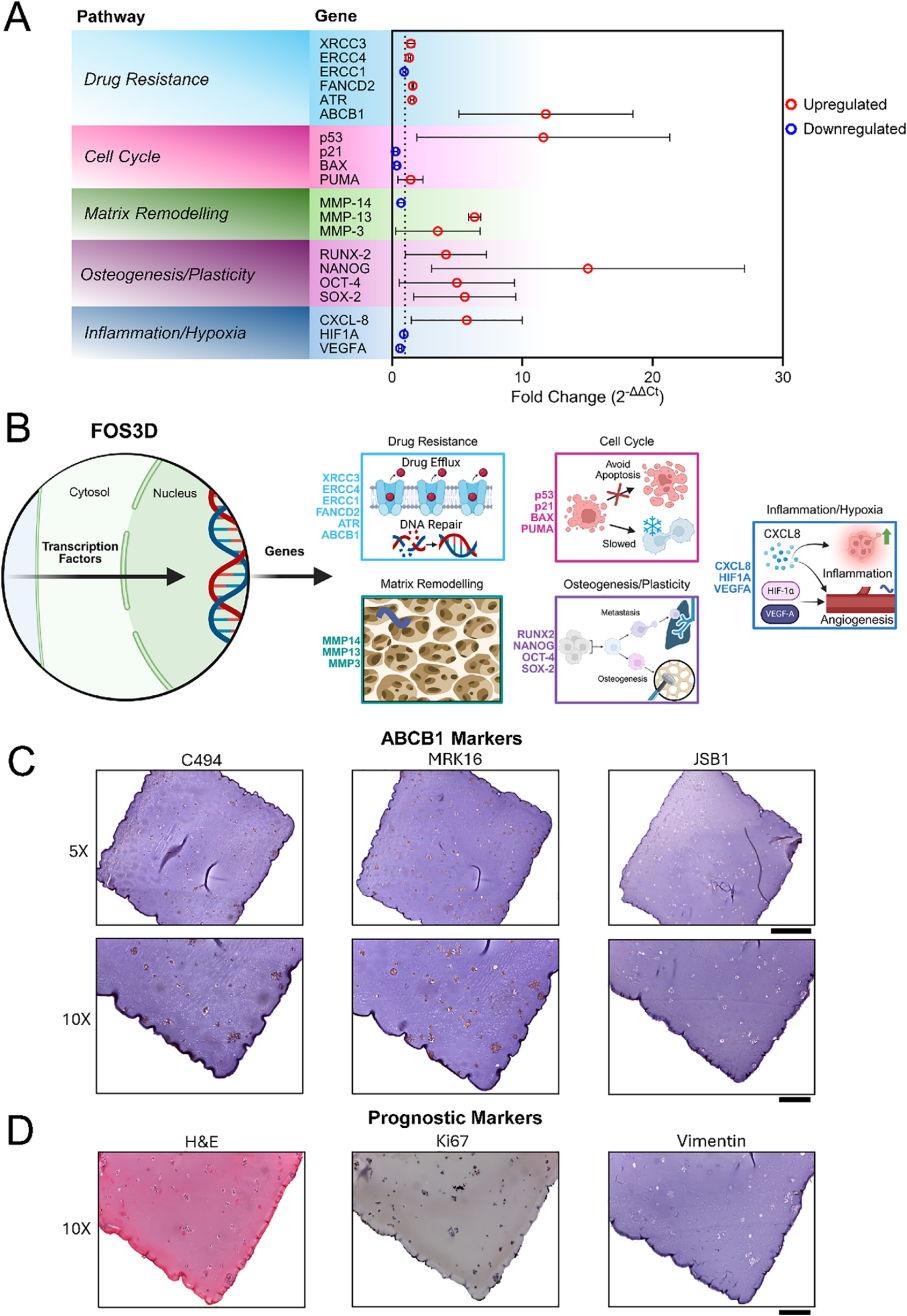
Transcriptional and prognostic profiling of MG‐63‐GFP cells cultured in FOS3D. (A) Relative mRNA expression of selected genes in MG‐63‐GFP cells cultured for 7 days in conventional 2D monolayers or in 3D GelMA hydrogels (FOS3D), measured by RT‐qPCR and expressed as 2^−ΔΔCt^ relative to 2D controls. The gene panel includes transcriptional markers related to drug resistance (XRCC3, ERCC4, ERCC1, FANCD2, ATR, ABCB1), regulation of cell‐cycle and apoptotic signaling (p53, p21, BAX, PUMA), ECM remodeling (MMP3, MMP13, MMP14), osteogenic and stemness‐associated transcription factors (RUNX2, NANOG, OCT4, SOX2), inflammatory signaling (CXCL8), and hypoxia‐associated pathways (HIF1A, VEGFA). Unpaired *t*‐tests were used to compare relative mRNA expression to 2D control, with statistically significant *p‐*values indicated on the graph, and all other results not statistically significant; data are presented as mean ± SEM n = 2 (GraphPad Prism, USA). (B) Schematic representation summarizing microenvironment‐associated signaling pathways detected in FOS3D constructs. The diagram integrates transcriptional changes associated with matrix remodeling, cell‐cycle regulation, inflammatory signaling, and lineage plasticity in MG‐63‐GFP cells cultured within GelMA hydrogels. (C) Immunohistochemical detection of the multidrug transporter ABCB1 (P‐glycoprotein) in MG‐63‐GFP FOS3D constructs using three independent antibody clones (C494, MRK16, and JSB1). Representative images are shown at 5× and 10× magnification (Leica, Germany). (D) Histological and prognostic marker characterization of MG‐63‐GFP FOS3D constructs. Hematoxylin and eosin staining shows cell distribution within GelMA hydrogels. IHC for Ki‐67 indicates proliferating cells, and vimentin staining confirms mesenchymal phenotype. Representative images are shown at 5× and 10× magnification (Leica, Germany). Scale bars: (C,D) 500 µm (5×), 200 µm (10×). Created in BioRender. Research, R. (2026) https://BioRender.com/d61iy7x.

To investigate molecular adaptations occurring within the FOS3D system, MG‐63‐GFP cells cultured for 7 days in GelMA hydrogels were analyzed using RT‐qPCR and IHC staining. Transcriptional analysis assessed gene expression across several functional categories including DNA repair and drug resistance, cell‐cycle regulation, ECM remodeling, osteogenic and stemness signaling, and hypoxia/inflammatory pathways. RT‐qPCR probe sequences listed in Table S1. Prognostic and resistance‐associated markers were further evaluated by IHC within the 3D constructs. RT‐qPCR analysis revealed differential gene expression patterns between MG‐63‐GFP cells cultured in FOS3D and those maintained in conventional 2D conditions (Figure 5A). Within the cell‐cycle regulatory network, p53 transcripts were elevated in 3D cultures, while downstream mediators showed variable expression. p21 was reduced relative to 2D controls, whereas BAX and PUMA displayed only modest changes. Analysis of ECM remodeling markers demonstrated increased transcription of several metalloproteinases (MMPs). MMP‐13 and MMP‐3 showed the most pronounced upregulation, while the membrane‐associated metalloproteinase MMP‐14 exhibited limited change. Genes associated with stemness and osteogenic differentiation were also upregulated in FOS3D cultures. NANOG demonstrated the strongest increase among the pluripotency markers, while OCT4 and SOX2 showed moderate elevation compared with 2D conditions. The osteogenic transcription factor RUNX‐2 was also increased in the 3D environment.

Evaluation of hypoxia and inflammatory signaling markers revealed modest changes. HIF‐1α remained near baseline expression, while VEGFA showed a non‐significant reduction relative to 2D cultures. In contrast, the pro‐inflammatory cytokine CXCL8 (IL‐8) demonstrated increased expression in the 3D system. A schematic overview summarizing the transcriptional pathways influenced by the FOS3D microenvironment is presented in Figure 5B, highlighting cellular processes including drug efflux and DNA repair, replicative stress responses, matrix remodeling, microenvironment signaling, and cell‐fate regulation. In addition, to offer translational relevance to FOS3D, we analyzed the expression of genes involved in DNA repair and chemoresistance. Specifically, the mRNAs of XRCC3, ERCC1, ERCC4, FANCD2, and ATR were partly increased in the 3D environment. More importantly, the prognostic biomarker and multidrug transporter ABCB1 demonstrated a variable but increasing trend in mRNA expression in the FOS3D system. To evaluate whether transcriptional changes were reflected at the protein level, IHC staining was performed on FOS3D sections targeting ABCB1 (P‐glycoprotein). Three independent antibody clones (C494, MRK16, and JSB1) were used to confirm staining reproducibility. Positive ABCB1 staining was detected throughout the constructs, appearing as a punctate brown signal distributed across the hydrogel sections (Figure 5C). The overall spatial pattern of staining was consistent across the three antibodies, although signal intensity varied between clones. Similar distributions of positive cells were observed at both 5× and 10× magnifications, indicating the presence of ABCB1‐expressing cells across the constructs. In parallel, histological examination using hematoxylin and eosin staining confirmed the presence of viable OS cell populations distributed within the GelMA scaffold (Figure 5D).

IHC staining for the proliferation marker Ki‐67 revealed scattered positive nuclei throughout the constructs, indicating the presence of actively cycling cells. Staining for vimentin, a mesenchymal intermediate filament protein commonly expressed in OS cells, demonstrated widespread cytoplasmic positivity within the samples. Comparable staining patterns were observed at 5× and 10× magnification.

Together, these findings demonstrate that MG‐63‐GFP cells cultured in the FOS3D system display measurable transcriptional adaptations and maintain expression of prognostic markers associated with proliferation, mesenchymal phenotype, and multidrug resistance.

### 2.6 Chemotherapeutic Drugs Screening in FOS3D

**FIGURE 6:**
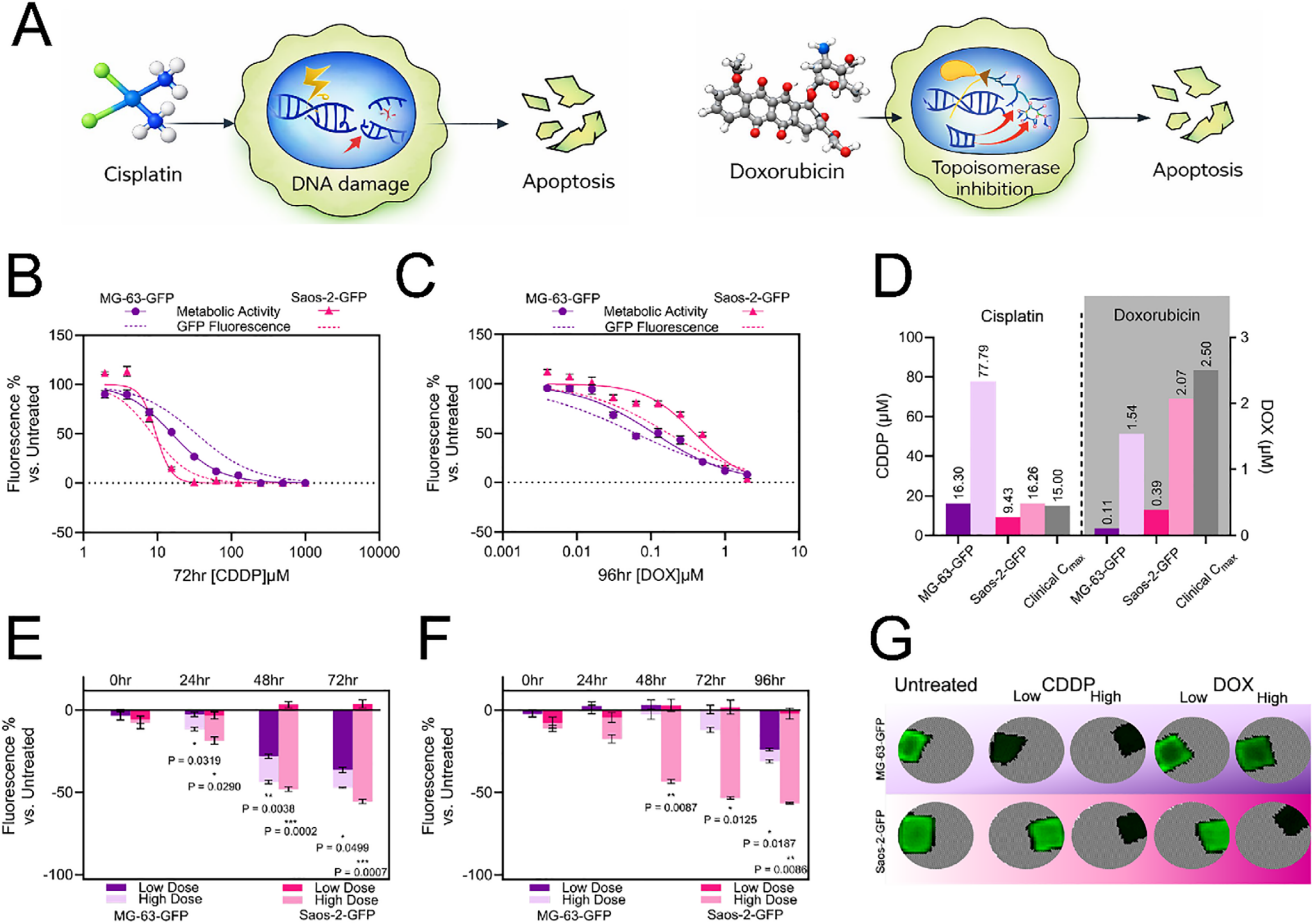
Chemotherapeutic drugs screening in FOS3D. (A) Schematic representation of primary CDDP and DOX mechanisms of action, illustrating DNA damage, topoisomerase inhibition, and apoptosis induction. (B,C) 2D drug studies demonstrating dose–response curves for CDDP and DOX treatment in MG‐63‐GFP and Saos‐2‐GFP monolayers. GFP fluorescence and metabolic activity show comparable four‐parameter logistic fits across increasing drug concentrations, validating fluorescence as a quantitative indicator of cytotoxicity. (D) IC_50_ (low dose) and IC_90_ (high dose) values derived from 2D metabolic assays for each cell line and each drug, compared with typical clinical plasma *C*_max_ ranges. These values were used to define experimental dosing in 3D assays. (E,F) 3D drug studies demonstrating longitudinal changes in GFP fluorescence in 3D constructs treated with Low‐ or High‐dose CDDP (E) or DOX (F). (G) Representative GFP well‐scan reconstructions of MG‐63‐GFP and Saos‐2‐GFP FOS3D treated with IC_50_ (low) or IC_90_ (high) concentrations of CDDP and DOX, showing dose‐dependent suppression of proliferation. Statistical analysis (E,F) comparing Low versus High Dose within each group was conducted using two‐way ANOVA with Tukey's multiple comparison test (Graphpad Prism, USA). Data are presented as mean ± SEM, *n* = 3 per [CDDP/DOX] (B,C), *n* = 3 per time point (E,F). CDDP = cisplatin, DOX = doxorubicin, GFP = green fluorescent protein, IC_50/90_ = inhibitory concentration %, *C*_max_ = Maximum plasma concentration.

Building on the ability of GFP fluorescence to monitor real‐time proliferation in both 2D and 3D environments, we next applied this approach to chemotherapeutic drugs screening using two biologically distinct OS cell lines: MG‐63‐GFP and Saos‐2‐GFP (Figure 6). These lines were selected to represent clinically relevant OS phenotypes. MG‐63 resembles low‐grade, fast‐growing, non‐metastatic early‐stage OS with wild‐type TP53 gene sequence but dysfunctional protein expression. Saos‐2, in contrast, represents a high‐grade, aggressive, TP53‐null OS subtype with slower proliferation and distinct drug‐response characteristics. Comparing these lines enables evaluation of drug sensitivity across a spectrum of clinically encountered OS behaviors. Validation of GFP fluorescence as a surrogate of cell viability was confirmed by metabolic activity assays for the Saos‐2‐GFP cell line (Figure S4). Because fluorescence intensity serves as the central quantitative readout of this screening approach, potential interference from drug‐intrinsic fluorescence was first evaluated. Cisplatin (CDDP) does not emit fluorescence, whereas Doxorubicin (DOX) exhibits an intrinsic excitation/emission spectrum centered at approximately 500/580 nm, which partially overlaps with the GFP detection window [33, 45]. To determine whether this spectral proximity could confound GFP‐based measurements, direct well‐scans were performed using GFP (470/515–520 nm) and DOX (500/580–630 nm) acquisition settings (Figure S5). Negligible signal was detected in the GFP channel in wells containing DOX alone, even at supraphysiological concentrations, while strong fluorescence was observed exclusively in the DOX channel. These findings demonstrate that DOX‐derived fluorescence does not meaningfully contribute to GFP signal within the experimental concentration range used, and that potential spectral bleed‐through is minimal and only becomes detectable at concentrations well beyond those employed in this study (Figure S5). Figure 6A summarizes the drug mechanisms and dose–response behavior of CDDP, a DNA crosslinking agent, and DOX, a topoisomerase II inhibitor. Dose–response curves generated from GFP fluorescence closely matched those obtained using metabolic assays for both cell lines in 2D (Figure 6B,C), confirming that reductions in GFP intensity reliably reflect drug‐induced cytotoxicity. IC_50_ and IC_90_ values derived from metabolic activity measurements (Figure 6D) demonstrated clear differences in drug sensitivity between cell lines. MG‐63‐GFP required higher concentrations of CDDP for equivalent growth inhibition, consistent with its known capacity to acquire CDDP resistance, while demonstrating greater sensitivity to DOX. Saos‐2‐GFP displayed moderate sensitivity to both drugs, consistent with its aggressive p53‐null genotype. These IC_50_‐equivalent (“Low Dose”) and IC_90_‐equivalent (“High Dose”) concentrations were subsequently applied to assess drug efficacy in FOS3D. When these clinically relevant concentrations were applied, longitudinal GFP reading (Figure 6E) and endpoint quantification of GFP fluorescence (Figure 6F,G) validated against metabolic and DNA content output (Figure S6) clearly demonstrated reduced drug susceptibility in 3D compared to 2D. Longitudinal GFP imaging (Figure 6E) demonstrated clear dose‐dependent cytotoxicity in both MG‐63‐GFP and Saos‐2‐GFP FOS3D. Untreated hydrogels displayed strong GFP signal, consistent with sustained proliferation and viability in 3D culture. In contrast, both CDDP and DOX reduced GFP fluorescence intensity and the apparent viable cell area, with high‐dose conditions producing the greatest suppression. In MG‐63‐GFP FOS3D, the IC_50_ dose of CDDP (16.3 µm) produced only moderate inhibition, whereas the IC_90_ dose (120 µm) strongly suppressed GFP fluorescence. DOX followed the same pattern, with the IC_50_ dose limiting expansion and the IC_90_ dose inducing pronounced cytotoxicity (Figure 6F). Saos‐2‐GFP FOS3D displayed similar behavior but with greater resistance to low‐dose treatments. For both CDDP and DOX, IC_50_ doses produced minimal inhibition, while IC_90_ concentrations caused significant reductions in fluorescence and metabolic viability (Figure 6G). Overall, the results revealed that drug concentrations highly effective in 2D monolayers are insufficient in FOS3D, whereas IC_90_‐equivalent doses consistently inhibit growth across both OS cell lines.

To further assess the clinical relevance of the dosing used in our 3D assays, experimental concentrations were compared with reported peak plasma levels (*C*_max_) in patients (Table 2). For CDDP, the IC_50_‐equivalent “Low Dose” for MG‐63‐GFP (16.3 µm) lies within the typical clinical range of approximately 10–20 µm, while the IC_90_‐equivalent “High Dose” (120 µm) exceeds circulating levels but likely reflects the additional diffusion barrier imposed by the 3D matrix. In Saos‐2‐GFP FOS3D, both the low (9.43 µm) and high (21.6 µm) CDDP doses fall at or near the clinical *C*_max_ window, indicating that the effective concentrations required to impact this high‐grade line are still physiologically attainable. For DOX, all IC_90_‐equivalent “High Dose” conditions remain comfortably within the reported clinical *C*_max_ range of roughly 1–5 µm. Collectively, these comparisons show that the enhanced drug tolerance observed in 3D arises from microenvironmental protection, and that our FOS3D operates within clinically meaningful exposure limits.

**TABLE 2:** Drug concentrations used in FOS3D and comparison with physiologically achievable patient plasma levels. Experimental CDDP and DOX concentrations used for MG‐63‐GFP and Saos‐2‐GFP drug screening, expressed in both micromolar (µm) and mass‐based (µg/mL) units, alongside reported typical clinical peak plasma concentrations (*C*_max_). Physiological range comments indicate the relationship between experimental doses and clinically relevant exposure levels, and the corresponding bibliography is reported in brackets.

Drug	Cell line	Experimental low dose (µm)	Low dose (µg/mL)	Experimental high dose (µm)	High dose (µg/mL)	Typical clinical *C*_max_ (µg/mL)	Physiological range comment
**Cisplatin (CDDP)**	MG‐63‐GFP	16.30	4.89	120	36	∼10–30 [46]	Low dose is in clinical range; high dose exceeds plasma but reflects diffusion requirements in 3D.
	Saos‐2‐GFP	9.43	2.83	21.60	6.48	∼10–30 [47]	Low dose slightly below plasma *C*_max_; high dose close to physiologic levels.
**Doxorubicin (DOX)**	MG‐63‐GFP	0.11	0.060	2.97	1.61	∼1–5 [48]	IC_90_ dose is well within clinical plasma levels.
	Saos‐2‐GFP	0.41	0.223	1.85	1.01	∼1–5 [48]	IC_90_ dose is comfortably within clinical range.

## 3 Discussion

FOS3D is a mechanically tunable GelMA‐based fluorescent osteosarcoma model that integrates GFP‐positive fluorescent cells as a practical and 3D method for tracking tumor proliferation and behavior longitudinally. In matrix‐based cultures, conventional readouts of proliferation and viability (e.g., metabolic conversion assays, DNA quantification, histology) typically require destructive sampling and extensive optimization to ensure assay penetration and complete hydrogel digestion. These constraints reduce throughput, necessitate large replicate numbers to reconstruct time courses, and prevent direct linkage between early phenotypic changes and later molecular endpoints within the same construct. The main methodological advance reported here is the ability to monitor fluorescence over time via multi‐well fluorescence reading, complemented by high‐resolution visualization using fluorescence microscopy techniques such as whole‐construct light‐sheet imaging. Together, these approaches reduce reliance on technically challenging and destructive endpoint analyses. More broadly, the findings herein align with the growing consensus that 3D cancer models can capture key microenvironmental constraints such as cell–ECM interactions, spatial confinement, diffusion gradients, and altered drug response, better than conventional 2D monolayers, thus enabling more informative preclinical drug screening pipelines [49].

By varying GelMA concentration and crosslinking duration, we generated variable stiffness values spanning ∼6–50 kPa, compatible with the reported Young's modulus values measured in the stromal phase of bone which is in the range of approximately 0.25–24.7 kPa [23], and demonstrated that these fabrication parameters can reproducibly modulate hydrogel mechanics while maintaining tumor cell viability. This is consistent with the well‐established tunability of GelMA through polymer content, degree of functionalization, and photo‐crosslinking conditions. Multiple recent reviews and optimization studies emphasize that relatively small adjustments in GelMA formulation and light exposure can produce substantial shifts in network density, stiffness, and transport properties, with downstream consequences for cell behavior [22, 50]. Findings regarding enhanced proliferation in softer hydrogels align with previous studies demonstrating stiffness‐dependent regulation of OS cell growth [51]. The selection of 6% GelMA with 2 min crosslinking duration provided an effective balance between mechanical integrity and biological permissiveness, supporting robust cell growth while enabling handling and longitudinal analysis [20, 22]. From a physiological perspective, this softer stromal‐like environment is particularly relevant, as OS cells interact extensively with compliant bone marrow niches, where mechanical confinement and ECM signaling contribute to tumor survival and therapy resistance [52]. In addition to matrix composition and stiffness, the cubic geometry of FOS3D offers practical advantages: cubes can be fabricated reproducibly, allow uniform cell seeding, and enable reliable mechanical testing under controlled compression conditions.

The strong correlation observed between GFP intensity and established proliferation metrics (DNA content and metabolic assays) supports endogenous fluorescence reading as an efficient surrogate readout for growth trends in both 2D and 3D. This is crucial because 3D matrices can complicate standard assays through penetration limits, incomplete lysis/digestion, and sampling bias; in contrast, fluorescence readouts can be acquired rapidly and repeatedly from intact constructs [24, 53]. Noticeably, there is a stronger correlation between endogenous fluorescence readings and DNA content analyses than metabolic activity. This highlights an important consideration, which is that endogenous fluorescence reading more closely measures the number of cells within a construct independently of the metabolic status of cells. This reinforces the use of orthogonal methods of biological assessment.

A practical consideration for the drug studies is optical interference: some compounds are intrinsically fluorescent or can quench signals, which can confound intensity‐based readouts [33]. In our model, we used DOX, one of the first‐line drugs for OS treatment, and its fluorescence is widely used for drug tracking, highlighting the need for appropriate filter selection, spectral separation, and experimental controls when combining fluorescence‐based proliferation assays with such treatments [33]. These considerations underscore the importance of orthogonal validation methods, including DNA quantification and imaging, particularly when screening chemically diverse drug libraries [54].

Paclitaxel was selected as the reference treatment in the validation experiments because it is a well‐established cytostatic chemotherapeutic agent widely used in tumor cell research to assess growth inhibition [55, 56]. The physiological mechanism of Paclitaxel involves the stabilization of microtubules leading to mitotic arrest, producing a clear, measurable reduction in proliferative output without inducing rapid apoptosis. Thus, Paclitaxel results in an ideal benchmark drug for testing the sensitivity, reliability, and dynamic range of GFP fluorescence as a proliferation readout in both 2D and 3D cultures. Drug sensitivity differed between culture formats, with Paclitaxel effects appearing earlier in 2D than in 3D cultures. The delayed onset of significant drug response observed in 3D constructs, which emerged around day 3, may be explained by cellular quiescence due to delayed growth kinetics under 3D conditions. Since anti‐cancer treatments such as Paclitaxel target mechanisms involved in division, slower proliferation rates can prolong this drug response and also contribute to decreased efficacy in 3D environments [57]. Additional environmental factors such as matrix‐mediated protection, diffusion barriers, and altered mechanotransduction could also contribute to this effect, further reinforcing the physiological relevance of the 3DFOS. The GelMA constructs display a dynamic mechanical behavior in the form of biodegradation over time. Initially, higher stiffness imposes physical resistance to cell spreading and expansion. Over time, hydrogel softening, likely driven by hydrolytic degradation and cell‐mediated remodeling, should create a progressively more permissive mechanical environment, characteristic of tumor progression in vivo, although longer time frames would be needed to observe this than examined here. While not investigated directly, this finding indicates the importance of mechanical and transport dynamics in regulating tumor growth and treatment response and further supports the value of the GelMA platform for modelling tumor biomechanics and therapeutic sensitivity.

The integration of light‐sheet microscopy significantly enhances the platform by enabling whole‐construct, spatially resolved imaging. Light‐sheet microscopy is particularly well suited to thick 3D samples, allowing rapid volumetric imaging with reduced photo‐bleaching and photo‐toxicity compared to point‐scanning techniques [58]. In this study, light‐sheet imaging and digital reconstruction revealed heterogenous cellular morphology across the hydrogel construct, with density‐dependent clustering and peripheral localization of cells. Cells under greater matrix confinement, in the central region of hydrogels, were subject to clustering, forming cellular aggregates reminiscent of spheroids. Whereas cells at the boundary or surface regions demonstrated extensive peripheral spreading over time. These patterns are consistent with tumor growth behavior in matrix‐based environments, where boundary/surface regions offer distinct mechanical and transport conditions, while central regions are subject to diffusion gradients and greater confinement. It is worth noting that since these observations are limited to MG‐63‐GFP cells and very specific matrix conditions, they are likely to vary depending on the cell type, seeding, and matrix stiffness and composition [4, 59, 60]. This could also be affected by the differentiation state and even co‐culture effects as noted in previous studies [61]. Nonetheless, the observed spatial patterns provide critical context for interpreting bulk fluorescence measurements and confirm that GFP monitoring captures meaningful biological changes rather than optical artefacts.

The transcriptional and prognostic profiling performed in the FOS3D system indicates that MG‐63‐GFP cells undergo coordinated molecular adaptations when cultured within the 3D GelMA microenvironment. These adaptations involve cell‐cycle regulation, ECM remodeling, stemness signaling, and multidrug resistance pathways, all of which are features commonly associated with OS progression. One notable change was the selective modulation of the p53 signaling axis. Although p53 transcripts were elevated in FOS3D, the downstream mediator p21 was reduced, while pro‐apoptotic markers such as BAX and PUMA remained relatively unchanged. This pattern is consistent with the p53‐mutant phenotype of MG‐63 OS cells, where transcriptional activation of p53 does not necessarily translate into canonical cell‐cycle arrest or apoptosis [62]. In MG‐63 cells, p21‐mediated apoptosis is typically triggered only under strong cellular stress conditions [63]. Instead, elevated p53 transcription can be interpreted as a marker of slowed replication kinetics and replicative stress in 3D, without activation of a strong apoptotic program which would include p21 induction. These findings align with the sustained viability observed in longitudinal fluorescence tracking and the maintenance of metabolic/DNA signals in 3D cultures, as well as the expression profile of hypoxia‐associated genes.

Alterations in ECM remodeling pathways further indicate that cells adapt to the surrounding hydrogel environment through dynamic matrix interaction. Increased expression of MMP‐13 and MMP‐3 suggests activation of matrix degradation processes associated with OS invasion. MMP‐13 targets fibrillar collagens, including types I, II, and III, whereas MMP‐3 degrades non‐fibrillar ECM components [64]. In contrast, the limited induction of the membrane‐bound collagenase MMP‐14 suggests that remodeling occurs primarily through diffuse matrix degradation rather than highly localized pericellular tunnelling. Such behavior may reflect the softer and more homogeneous mechanical environment of the GelMA scaffold compared with mineralized bone matrices. The transcriptional profile also revealed clear induction of stemness‐associated transcription factors, including NANOG, OCT4, and SOX2, together with increased expression of the osteogenic regulator RUNX‐2. Stemness markers are frequently associated with tumor plasticity, metastatic potential, and increased resistance to therapy [65, 66]. RUNX‐2, in contrast, regulates osteoblast differentiation and bone ECM production [67]. The simultaneous expression of stem‐like and osteogenic transcriptional programs suggests that cells within FOS3D occupy a transcriptionally plastic state, capable of transitioning between proliferative, invasive, and lineage‐associated phenotypes. Importantly, RUNX‐2 is also known to respond to mechanical cues and can regulate MMP‐13 expression in response to matrix stiffness, thereby facilitating cellular adaptation to changing extracellular environments [68]. Overall, these results suggest that encapsulation in GelMA does not force cells down a single cell‐fate but rather supports intra‐tumoral heterogeneity by keeping cells in a perpetual adaptive state between proliferative, invasive, and osteogenic programs. This forms the basis for a robust model of an early‐state aggressive and metastatic OS with cancer stem cell properties.

Hypoxia‐associated signaling was comparatively modest in the FOS3D constructs. HIF‐1α expression remained close to baseline, while VEGFA showed a slight decrease relative to 2D culture. These findings indicate that the hydrogel constructs likely experience only mild oxygen limitation under the present culture conditions, consistent with the relatively small construct dimensions and the diffusion‐permissive nature of GelMA. Instead of reproducing a strongly hypoxic tumor core, the transcriptional profile may reflect an early tumor microenvironment characterized by inflammatory signaling. In this context, the observed increase in CXCL8 (IL‐8) may be particularly relevant. CXCL8 has been implicated in OS progression through its ability to stimulate angiogenesis, activate matrix remodeling pathways, and reinforce stemness signaling through interactions with OCT4, SOX2, and NANOG [69, 70, 71].

A key observation in this study was the upregulation of ABCB1 and the detection of its protein product, P‐glycoprotein, throughout the FOS3D constructs. ABCB1 encodes an ATP‐dependent drug efflux transporter that plays a major role in chemotherapy resistance in OS. In clinical practice, IHC assessment of P‐glycoprotein is often performed on diagnostic biopsy specimens before treatment to evaluate potential drug resistance and support therapeutic stratification. Clinical studies have demonstrated that P‐glycoprotein expression can stratify high‐grade OS patients and correlate with treatment outcomes [34, 36]. Earlier work has also identified a relationship between P‐glycoprotein expression and p53 status in OS, suggesting a possible interaction between cell‐cycle regulation and multidrug resistance pathways [35]. Beyond intrinsic transporter expression, multidrug resistance in OS can also arise through microenvironment‐mediated mechanisms, including the intercellular transfer of resistance determinants via extracellular vesicles and exosomes [72]. Experimental xenograft models using ABCB1‐overexpressing MG‐63 variants have further demonstrated that OS cells can maintain multidrug‐resistant phenotypes in vivo [73]. The presence of ABCB1 expression within the FOS3D constructs therefore indicates that the model captures an important component of therapy‐relevant tumor biology.

Importantly, the transcriptional and prognostic signatures identified in the FOS3D system anticipated the functional behavior observed in the subsequent drug‐screening experiments. The combination of stemness signaling, ECM remodeling, and ABCB1‐associated drug efflux pathways is consistent with microenvironment‐mediated protection from chemotherapeutic agents. Indeed, the chemotherapeutic screening performed in Section 2.6 demonstrates that concentrations of CDDP and DOX that effectively suppress proliferation in 2D monolayer cultures are markedly less effective in the FOS3D constructs. This reduced drug susceptibility supports the interpretation that the 3D constructs confer microenvironment‐dependent drug tolerance, a phenomenon widely observed in solid tumors [49, 74, 75]. Together, these findings indicate that the FOS3D platform reproduces several biological hallmarks relevant to OS progression and therapy response. By supporting transcriptional plasticity, matrix remodeling behavior, and multidrug resistance signaling, the model provides a physiologically relevant framework for investigating tumor–microenvironment interactions and for evaluating therapeutic strategies in a context that more closely resembles the in vivo tumor environment.

Drug response experiments using frontline OS chemotherapeutics, CDDP and DOX, further highlighted the translational relevance of the platform. Reduced drug sensitivity in 3D relative to 2D is widely reported and attributed to ECM protection, diffusion limitations, and altered cell states [49, 76]. Our findings support this notion, with higher drug doses required to achieve comparable inhibitory effects in 3D constructs compared to 2D. Moreover, these doses were closer to clinical *C*_max_ values. This reinforces the value of engineered 3D systems as intermediate screening platforms that better predict therapeutic efficacy than 2D models. DOX response in MG‐63 cells warrants particular consideration. Although this drug induces DNA damage and apoptosis, MG‐63 cells harbor dysfunctional p53 signaling, meaning apoptosis may occur through p53‐independent pathways. Indeed, DOX has been shown to induce apoptosis via alternative mechanisms including oxidative stress and mitochondrial pathways independent of p53 activation [77]. This may contribute to variable drug sensitivity and reflects the complexity of OS therapeutic response.

Admittedly, this study has several limitations. The use of two established OS cell lines (MG‐63‐GFP and Saos‐2‐GFP), although widely adopted in OS research, does not capture the full clinical heterogeneity of the disease in terms of genetic background, differentiation state, osteoid production, and therapeutic response. Moreover, cell lines transfected to express GFP can display different phenotypic characteristics compared to parent cell lines [78]. Importantly, however, the core technical features of the platform, including stable GFP expression, linear scaling of fluorescence with cell number, and strong correlation between GFP signal and orthogonal proliferation metrics, were reproducibly demonstrated across both lines, supporting the robustness and line‐agnostic nature of the measurement strategy. This cross‐validation suggests that the workflow is transferable rather than tailored to a single cellular model. Nevertheless, expanding the system to include additional OS cell lines and, critically, patient‐derived tumor cells will be an essential next step to better capture inter‐patient variability and to strengthen translational relevance [79].

A second limitation is that the current model represents a simplified tumor microenvironment focusing primarily on a single hydrogel ECM component without incorporating bone‐specific mineral phases, additional stromal, immune, or vascular cell populations, or dynamic factors such as interstitial fluid flow, compression and torsion [6, 20]. While this reduces confounding variables and can facilitate a mechanistic understanding of cell behavior, it cannot be considered equivalent to more complex systems or models which feature factors present in the in vivo OS microenvironment. Despite this, GelMA does provide a biologically relevant, tunable matrix supporting cell adhesion, spreading, and mechanotransduction and could form the basis of future iterations incorporating composite hydrogels containing hydroxyapatite or other calcium phosphate phases, spatially patterned stiffness, multicellular co‐cultures, and even dynamic perfusion‐based or cyclic compression systems to better emulate features currently absent in the presented model [4, 80]. In parallel, integrating readouts of matrix remodeling and osteogenic activity (e.g., collagen deposition, alkaline phosphatase activity, or mineralization markers) would further strengthen biological relevance without compromising the scalability of the GFP‐based monitoring approach. However, increasing mineral content and structural complexity may introduce additional technical challenges, including reduced optical transparency and increased light scattering, which could limit fluorescence accessibility and detection sensitivity within dense or opaque mineralized regions [81]. For example, hydroxyapatite, when used in greater concentrations, can cause opacity, which end‐point techniques are not compatible with our longitudinal monitoring technique, such as micro‐CT, optical clearing, or histological staining. However, alternatives exist by designing porous constructs or including hydroxyapatite in other forms such as nano‐hydroxyapatite or magnesium‐doped hydroxyapatite, which can preserve optical clarity, enhance mechanical properties, and even facilitate osteogenic differentiation [82, 83, 84]. Therefore, care should be taken to when selecting matrix composition, in optimizing imaging parameters, and deciding assays or other quantification strategies.

Taken together, the FOS3D is robust because it couples a tunable hydrogel system with a fluorescence monitoring strategy that is both scalable (multi‐well fluorescence intensity reading) and information‐rich (volumetric microscopy), while retaining compatibility with standard destructive assays and molecular profiling. The most important next steps for increasing translational relevance would be expanding to patient‐derived OS cells to derive broader phenotypic and molecular profiles, and incorporating microenvironmental complexity (e.g., stromal/immune/endothelial components and/or mineral gradients), while retaining the same 3D monitoring backbone; these directions are frequently identified as key to improving predictive power of 3D cancer models for drug development.

## 4 Conclusion

In this study, we developed and validated a mechanically tunable GelMA‐based, fluorescent 3D OS model (FOS3D) that enables real‐time, 3D monitoring of tumor proliferation using endogenous GFP fluorescence. By modulating GelMA concentration and photo‐crosslinking duration, we generated constructs spanning a clinically relevant OS stromal phase stiffness range while maintaining tumor cell viability. Across both 2D and 3D formats, endogenous GFP fluorescence correlated strongly with conventional proliferation endpoints, confirming GFP‐based monitoring as a reliable surrogate metric for longitudinal growth assessment without construct disruption. Importantly, coupling GFP monitoring with light‐sheet microscopy enabled whole‐construct, high‐content visualization and quantification of cell distribution, number, and morphology over time, revealing spatiotemporal patterns such as progressive clustering and increased spreading at the hydrogel periphery. When applied to chemotherapy screening, GFP‐based readouts captured dose‐dependent responses to CDDP and DOX in two distinct OS cell lines and demonstrated the expected microenvironment‐driven drug tolerance in 3D relative to 2D. Finally, RT‐qPCR profiling confirmed that 3D culture elicited transcriptional adaptations beyond proliferation and favoring a stem‐like state with osteogenic lineage activity characteristic of an aggressive OS and further supporting the biological dynamism of the engineered microenvironment. Furthermore, FOS3D allowed the expression and IHC detection of ABCB1/P‐glycoprotein, an important prognostic biomarker currently used to stratify patients for treatment, particularly in clinical studies aimed at identifying high‐risk patients who might benefit from modified chemotherapy regimens [36].

Collectively, these findings support the integration of 3D fluorescence tracking into 3D tumor platforms as a scalable strategy to quantify OS growth and treatment response over time while preserving samples for downstream analyses. This approach can accelerate iterative optimization of OS models and improve the throughput and translation of preclinical drug screening in physiologically relevant 3D conditions.

## 5 Experimental Section

### 5.1 Materials

Gelatin methacryloyl (GelMA), ANFF (Wollongong, NSW, Australia), DOF calculated by NMR 83% ± 4%; Lithium phenyl‐2,4,6‐trimethylbenzoylphosphinate‐LAP, Merck, St. Louis, MO, USA); SYLGARDTM 184 polydimethylsiloxane (PDMS) (Dow, USA); Dulbecco's Phosphate Buffered Saline (PBS 1X), Merck (St. Louis, MO, USA); Penicillin‐Streptomycin (Pen/Strep) (10 000 U/mL), Gibco (Waltham, MA, USA); *L*‐Glutamine 200 mm, GIBCO (Grand Island, NY, USA); HEPES buffer (1 m), GIBCO (Grand Island, NY, USA); Dulbecco's modified Eagle's glucose (4500 mg/L), #D6046, Sigma‐Aldrich (St. Louis, MO, USA); McCoy's 5A (Modified) Medium (Thermo Fisher Scientific); McCoy's 5A Media HyClone no phenol red (Cytiva, USA); Dulbecco's modified Eagle's glucose (4500 mg/L) no phenol red (Gibco, Thermo Fisher Scientific); Trypsin‐EDTA (0.25%) phenol red (Thermo Fisher Scientific Australia Pty Ltd); Cisplatin‐S4 #D3371 (TCI‐CHEMICALS); DOXORUBICIN HYDROCHLORIDE #D1515 (Sigma); PACLITAXEL #P1632 (Tokio Chemical Industry CO, Tokio, Japan); Quant‐iT PicoGreen dsDNA Assay Kit #P7589 (Thermo Fisher Scientific); Cell Titer‐Blue Viability kit (Promega, Madison, WI, USA); Paraformaldehyde (Santa Cruz Biotechnology, Dallas, TX, USA); 4′,6‐Diamidine‐2′‐phenylindole dihydrochloride (DAPI) #D1306 (Roche); Dimethyl sulfoxide ACS reagent, ≥99.9%‐DMSO #472301 (Merck).

### 5.2 Cell Culture

Two human OS cell lines, MG‐63 and Saos‐2, originally purchased from ATCC (MG‐63, CRL‐1427; Saos‐2, HTB‐85), were used in this study. Cells were genetically labelled to enable constitutive cytoplasmic fluorescence and longitudinal quantification. Briefly, OS cells were lentiviraly transduced using Firefly luciferase + eGFP Lentifect purified lentiviral particles (GeneCopoeia; Cat. No. LPP‐hLUC‐Lv201) containing a CMV promoter driving humanized firefly luciferase (hLUC) and an SV40 promoter driving GFP, with puromycin resistance (Pac gene) bicistronicaly co‐expressed with GFP to enable stable selection. Following transduction, stable GFP‐positive populations were enriched by fluorescence‐activated cell sorting (multiple rounds where required) to increase expression homogeneity. For further stabilization of GFP expression, cells were maintained under puromycin selection (2 µg/mL), which yielded near‐homogeneous GFP‐positive populations compared to non‐selected cultures. Puromycin was not present during the experiments presented in the current study. Cryopreserved vials of MG‐63 GFP/Firefly luciferase and Saos‐2 GFP/Firefly luciferase were supplied as early‐passage stocks and used for expansion and experimental work [39]. Cells were maintained in T175 cm^2^ flasks and incubated at 37°C, 5% CO_2_, with media replenished every 2, 3 days. MG‐63‐GFP cells were cultured in high‐glucose DMEM supplemented with 10% fetal bovine serum (FBS), 1% GlutaMAX, and 1% penicillin/streptomycin. Saos‐2‐GFP cells were cultured in McCoy's 5A medium supplemented with 10% FBS, 1% GlutaMAX, and 1% penicillin/streptomycin. Cells were passaged at 80%–90% confluency, and all experiments were performed using cells within ≤20 passages to minimize phenotypic drift.

### 5.3 Two‐Dimensional Model

For 2D experiments, GFP‐OS cells were detached using Trypsin, counted using an automated cell counter (Countess, Thermo Fisher Scientific), and seeded into standard tissue‐culture‐treated 96‐well plates (Greiner, Germany). Cells were cultured in 200 µL complete growth medium per well and maintained at 37°C, 5% CO_2_ for the duration of experiments. Media was replaced every day unless otherwise stated. In Figure 2B,D, different cell densities were generated by creating a serial dilution, starting with 80 000 cells and decreasing by half with each successive dilution up to 5000 cells. Whereas in Figure 2F,H, a fluorescence‐based longitudinal study, cells were seeded at 10 000 cells/well (equivalent to **∼**1600 cells/cm^2^) and GFP fluorescence was monitored over time using a plate reader (see Section 5.6). Where drug treatments were applied, dosing was performed after cell attachment (typically 18–24 h post‐seeding), and fluorescence readings were acquired at defined time points.

### 5.4 Fluorescence‐Enabled Tri‐Dimensional Model (FOS3D)

Gelatin‐methacryloyl (GelMA) synthesized and provided by ANFF (Wollongong, NSW, Australia) was sterilized using ethylene oxide (EtO) gas at room temperature. GelMA was dissolved to several final concentrations (60, 80, and 100 mg/mL) in sterile PBS (1×) containing penicillin (100 U/mL) and streptomycin (100 µg/mL) (reported throughout the manuscript as 6% GelMA, 8% GelMA, 10% GelMA). The photo‐initiator lithium phenyl‐2,4,6‐trimethylbenzoylphosphinate (LAP) was prepared as a 1% (w/v) stock solution in PBS (1×), sterile filtered through a 0.22 µm syringe filter, and used at a final working concentration of 0.06% (w/v).

Biofabrication of the 3D model was achieved by casting 35 µL GelMA mixed with LAP and OS cells into silicone molds and photo‐crosslinking using a LunaCrosslinker (Gelomics; now distributed by BioNordika, Oslo, Norway) at 405 nm and 9–10 mW/cm^2^ for 1, 2, and 3 min. A schematic of the biofabrication workflow is shown in Figure 1A. Mold dimensions were 3.3 × 3.3 × 3.3 mm (total volume 35–36 mm^3^), requiring 35 µL of hydrogel precursor containing the desired cell number. Constructs were rinsed twice in PBS (1×) and cultured in an ultra‐low attachment 96‐well plate (Corning) in 200 µL culture medium per well at 37°C and 5% CO_2_, with medium changes performed daily. In Figure 2C,E, different cell densities were generated by creating a serial dilution, starting with 80 000 cells and decreasing by half with each successive dilution up to 5000 cells. In Figure 2G,I, an initial seeding number of 70 000 cells per hydrogel was used. Paxlitaxel was used at 0.117 mm (0.1 mg/mL) as previously established in our studies [85]. Cells were seeded in both 2D and 3D cultures and treated with 200 µL of paclitaxel diluted in complete culture medium for 7 days, with medium replacement on days 1, 2, and 4.

To establish comparable seeding densities between 2D and 3D studies for the drug studies (Section 5.10), the average theoretical distance between cells was calculated using Equations (1) and (2), assuming a perfectly homogeneous distribution across either a 2D plane or 3D cube. These calculations do not account for cell volume and therefore represent the distance between the centers of adjacent cells.

(1) $$\mathrm{center}-\mathrm{to}-\mathrm{center}\;\mathrm{distance}=\frac{1}{\sqrt[{2}]{\mathrm{cell}\;\mathrm{density}\;(\mathrm{cells}/cm^{2})}}$$

(2) $$\mathrm{center}-\mathrm{to}-\mathrm{center}\;\mathrm{distance}=\frac{1}{\sqrt[{3}]{\mathrm{cell}\;\mathrm{density}\;(\mathrm{cells}/cm^{3})}}$$

This equates to a center‐to‐center distance of approximately 70 µm for a seeding number of 5000 cells/well and 70 000 cells/hydrogel in 2D and 3D, respectively.

### 5.5 Mechanical Testing

Compression of hydrogels was performed using a TA Electroforce 5500 mechanical loading device (TA Instruments, New Castle, DE) fitted with a 250 g load cell. Hydrogels were mounted to a glass slide and compressed to 30%–40% strain at a rate of 0.01 mm/s. Microscopy images were taken prior to testing to allow for calculation of the cross‐sectional area of each sample using ImageJ software (NIH, USA), then used to calculate the compressive Young's modulus across the 10%–15% strain range.

### 5.6 Fluorescence Monitoring of Cells in 2D and 3D

Three‐dimensional, real‐time imaging and automated fluorescence mapping readings were performed longitudinally to assess cell viability, proliferation, and responses to cytotoxic drug treatment. GFP expression enabled real‐time, in situ monitoring via fluorescence plate reading and microscopy. For these experiments, phenol red–free cell culture medium was used to eliminate background autofluorescence originating from phenol red. Fluorescence was quantified using a CLARIOstar Plus microplate reader (BMG LABTECH), measuring GFP excitation/emission at 470–15/515–20 nm in both 2D and 3D cultures plated in 96‐well plates containing 200 µL phenol red–free medium or PBS (1×). Optical settings were: dichroic filter 493.8 nm, gain 1000, and focal height 4 mm. Well‐scan acquisition was performed using 8 flashes per scan point, a 30 × 30 scan matrix map, and a 6 mm scan width. Background subtraction of 1000 a.u is used to exclude the region surrounding the hydrogel and isolate quantification to average fluorescence values within the hydrogel and not the surrounding area. For 2D studies, background subtraction was not necessary since flat monocultures covered the well‐plate surfaces.

### 5.7 Cells Viability Assays

#### 5.7.1 DNA Quantification

Hydrogels were digested in 500 µL of Papain buffer overnight at 60°C. After 10 000 g centrifugation for 10 min at room temperature, DNA‐containing supernatant was collected in a new Eppendorf tube. The protocol was followed for the PicoGreen double‐stranded DNA assay, including producing a standard curve with lambda‐DNA, and excitation was read at 530 nm (CLARIOstar Plus microplate reader).

#### 5.7.2 Metabolic Activity

Culture media were replaced with 200 µL of Cell Titer‐Blue (Promega) reagent in a 1:5 ratio with phenol‐free culture media. After 2‐hour incubation, fluorescence was measured at 590 nm emission using a CLARIOstar Plus microplate reader.

### 5.8 Light‐Sheet Microscopy

Hydrogels were placed in a 24‐well plate and fixed in 1% paraformaldehyde for 4 h under gentle shaking, then washed and stored in PBS. The DAPI staining was performed by diluting the stock solution of 1 mg/mL to a 1:100 working concentration, then adding 1 mL to each sample in a 1.5 mL Eppendorf tube and incubating at room temperature for 2 h with gentle shaking. During acquisition, cytoplasmic GFP fluorescence was imaged using the 510 nm laser, while cell nuclei stained using DAPI were imaged using the 405 nm laser. A light‐sheet microscope was used to capture and render optical sections of cell‐laden hydrogels (Miltenyi Biotec, Germany, UltraMicroscope Blaze). The hydrogels were attached to the light‐sheet arm using cross‐linkable glue and lowered into a reservoir containing glycerol imaging solution. Samples were located and their height and area defined to facilitate automated acquisition via UltraMicroscope Platform software. Images were converted and analyzed using Imaris 10 software (Oxford Instruments, England).

Surface volumes were generated using the Surface function in Imaris 10. The input diameter was set to 20 µm, and thresholding was based on the maximum signal intensity from GFP cells in order to remove background noise. To account for overlapping signal from adjacent cells, the software's Object Splitting function was used, and a seeding number of 15 µm, the average diameter of a cell, was applied to help distinguish individual cells. Object statistics were gathered based on these generated surfaces, including sphericity measurement. Sphericity was a measure of how closely a cell resembles a sphere. According to Wadell's definition, which we have adapted for the purpose of investigating cells, sphericity was defined as the ratio of the surface area of a sphere, with the same volume as the cell, to the actual surface area of the cell [86].

$$\Psi =\frac{\frac{1}{\pi^{3}}{\left(6V_{C}\right)}^{\frac{2}{3}}}{A_{C}}$$

where *V*_C_ = volume of the cell, *A *= surface area of the cell.

Spots, like the Surface function, were generated in Imaris 10 using an input diameter of 15 µm for the DAPI signal, and 20 µm for the GFP signal. Thresholding was once again based on maximum signal intensity in both channels. Object Statistics were determined, allowing spots to be counted per channel.

### 5.9 RT‐qPCR

Total cellular RNA was extracted from MG‐63‐GFP cells cultured in 2D and in FOS3D after 7 days of culture. At the end of each experiment, up to 3 gels were freeze‐dried in liquid nitrogen, pulverized through mortar and pestle, and resuspended in up to 1 mL of TRIzol Reagent (Thermo Fisher Scientific). Samples were kept frozen at −20°C until RNA extraction. Lysis was performed following the instructions of TRIzol Reagent guidelines, and once obtained, the aqueous phase containing RNA was mixed with 1 volume of 70% absolute ethanol and transferred to an RNeasy Mini Spin column (RNeasy Mini Kit, Qiagen). Therefore, the RNA extraction was carried out following the manufacturer's instructions. Elution volume was 30 µL of RNase‐free water, and 1 µL was used to quantify RNA through Nanodrop ONE (Thermo Fisher Scientific). Five hundred nanograms of RNA were reverse transcribed using iScript cDNA Synthesis Kit (Bio‐Rad Laboratories). The obtained cDNA was diluted 1:10 in Ultrapure distilled water, DNase‐RNase‐free (Invitrogen), and 1 µL was used for gene expression analyses (reaction volume 10 µL). The real‐time PCR CFX OPUS 96 detection system (Bio‐Rad Laboratories) was used to perform the analyses. Both SYBR Green (SsoAdvanced Universal SYBR Green Supermix; Bio‐Rad Laboratories and TAQMAN approaches (SsoAdvanced Universal Probes Supermix; Bio‐Rad Laboratories) were employed. All the information regarding primers and probes is listed in Table S1. Data were analyzed through the 2^−ΔΔCt^ method, and β‐actin was used as a housekeeping gene [87]. Results are shown as a ratio referred to 2D cultivated cells.

### 5.10 Histology and Immunohistochemistry of FOS3D Constructs

FOS3D hydrogel constructs containing MG‐63‐GFP cells were collected after 7 days of culture and processed for histological and immunohistochemical analysis. Constructs were rinsed in phosphate‐buffered saline (PBS1X) and fixed in 10% paraformaldehyde for 24 h at room temperature. Following fixation, samples were dehydrated through a graded ethanol series, cleared in xylene, and embedded in paraffin using standard histological procedures. Paraffin blocks were sectioned into 2 µm thick sections using a rotary microtome and mounted onto positively charged glass slides. For routine histological evaluation, sections were deparaffinized in xylene, rehydrated through graded ethanol solutions, and stained with hematoxylin and eosin (H&E) following standard protocols using an automated slide stainer (BenchMark Ultra, Ventana Roche).

For ABCB1 and Vimentin IHC, sections were first deparaffinized and rehydrated. Endogenous peroxidase activity was quenched by incubation with 3% hydrogen peroxide for 30 min in methanol. Non‐specific binding was blocked using 2% normal horse serum in PBS (1×) for 15 min at room temperature. Sections were then incubated overnight at 4°C with primary antibodies diluted in blocking buffer. The multidrug resistance transporter ABCB1 (P‐glycoprotein) was detected using three independent monoclonal antibody clones: (1) C494, Mouse anti‐ MDR1 P‐gp Clone C494 (187243, Invitrogen) 1:100; (2) MRK16, Mouse anti‐MDR1 P‐gp Clone MRK16 (MBS488127, My Biosource) 1:300; and (3) JSB1, mouse anti‐MDR1 P‐gp Clone JSB1 (MON9011P, Monosan) 1:80. Vimentin primary antibody (monoclonal Mouse Anti‐Vimentin Clone V9, M0725, DAKO, Agilent Technologies) was diluted 1:50 in 2,5% v/v normal serum in PBS (1×). The day after, following PBS washes, sections were incubated with an anti‐mouse biotinylated secondary antibody for 30 min at room temperature, followed by incubation with the avidin–biotin peroxidase complex (ABC) for an additional 30 min (VECTASTAIN ABC‐HRP Kit, Peroxidase Mouse IgG, Vector Laboratories). After washing with PBS, sections were incubated with horseradish peroxidase (HRP)‐conjugated secondary antibodies for 1 h at room temperature. Signal was visualized using 3,3′‐diaminobenzidine (DAB) chromogenic substrate, producing a brown precipitate at sites of antigen localization. Sections were counterstained with hematoxylin, dehydrated, cleared, and mounted with permanent mounting medium. Bright‐field images were acquired using an optical microscope at 5× and 10× magnification (Leica, Germany). Additional staining was performed for Ki‐67 to assess proliferative activity of OS cells. A primary antibody anti‐rabbit anti‐Ki67 was used, and the staining was performed through an automatic slide stainer (BenchMark Ultra, Ventana Roche, UltraView Universal DAB Reveal Kit).

### 5.11 Drug Studies

Two drugs were used: Cisplatin (CDDP) and Doxorubicin (DOX). These constitute the typical frontline chemotherapeutic treatment of OS. Stock solutions (10 mm) were created by dissolving CDDP powder in PBS (1×), and DOX in DMSO (1 m) and PBS (1×) in a 1:1 ratio. These solutions were filtered with a sterile 0.22 µm filter (Millex). The products were used immediately or stored in −20°C for up to 1 month.

During experimentation, twofold serial dilutions were prepared in cell culture media, with concentrations of DOX (0–2 µm), and CDDP ranging (0–1 mm).

Two‐dimensional drug studies were conducted with a seeding number of 20 000 cells per well in a 96‐well plate (6400 cells/cm^2^) to achieve close to confluent plates prior to dosing. Tri‐dimensional studies were conducted with a seeding number of 2 × 10^6^ cells/mL, or 70 000 cells per 35 µL hydrogel in a 96‐well ultra‐low attachment plate.

The relative half‐maximal inhibitory concentration (IC_50_), defined as the point at which metabolic activity was reduced by half compared to untreated cells, was used to assess drug sensitivity between cell lines. The response of cells to different drug concentrations is defined by a four‐parameter logistic regression, which fits a sigmoidal curve based on the upper and lower asymptotes according to the four‐parameter logistic regression equation:

$$y=d+\frac{a-d}{1+\left(\frac{x}{c}\right)b}$$

where *a* = lower‐asymptote, *d* = upper‐asymptote, *c *= IC_50_, *b* = Hill Slope.

### 5.12 Statistical Analysis

Data were reported as a mean with error bars representing standard error of the mean. All statistical analysis was performed using GraphPad Prism 10 software (GraphPad, USA). Details of the statistical analysis techniques used are included in figure descriptions.

## Author Contributions

**William Humble** carried out methodology, experimental procedures, analyses, data curation, and writing of the original draft. **Wiktor Zywicki** carried out initial methodology, investigation, and review of the manuscript. **Enrico Lucarelli** helped with methodology, experimental procedure consultations, and review of the manuscript. **Ania Naila Guerrieri** carried out RT‐qPCR experimental procedures and analyses and review of the manuscript. **Francesca Taraballi** provided the GFP‐transfected cell lines. **Gianluca Cidonio** helped with methodology, experimental procedure consultations, and review of the manuscript. **Claudia Di Bella** reviewed the manuscript and provided clinical perspective throughout the whole project. **Carmine Onofrillo** provided intellectual input and extensively reviewed the manuscript. **Andrea J. O'Connor** carried out conceptualization, resources, supervision, and review of the manuscript. **Serena Duchi** carried out conceptualization, methodology, investigation, resources, supervision, project administration, writing & editing of the manuscript. All authors have read and reviewed the manuscript and agree on the final curated version.

## Conflicts of Interest

The authors declare no conflicts of interest.

## Supporting information




**Supporting File 1**: advs76031‐sup‐0001‐SuppMat.docx

**Supporting File 2**: advs76031‐sup‐0002‐MovieS1.mp4.

**Supporting File 3**: advs76031‐sup‐0003‐MovieS2.mp4.

**Supporting File 4**: advs76031‐sup‐0004‐MovieS3.mp4.

**Supporting File 5**: advs76031‐sup‐0005‐MovieS4.mp4.

## Data Availability

The data that support the findings of this study are available from the corresponding author upon reasonable request.

## References

[1] H. Page , P. Flood , and E. G. Reynaud , “Three‐Dimensional Tissue Cultures: Current Trends and Beyond,” Cell and Tissue Research 352 (2013): 123–131, 10.1007/s00441-012-1441-5. 22729488

[2] D. Wirtz , K. Konstantopoulos , and P. C. Searson , “The Physics of Cancer: The Role of Physical Interactions and Mechanical Forces in Metastasis,” Nature Reviews Cancer 11 (2011): 512–522, 10.1038/nrc3080. 21701513 PMC3262453

[3] F. Ruedinger , A. Lavrentieva , C. Blume , I. Pepelanova , and T. Scheper , “Hydrogels for 3D Mammalian Cell Culture: A Starting Guide for Laboratory Practice,” Applied Microbiology and Biotechnology 99 (2015): 623–636, 10.1007/s00253-014-6253-y. 25432676

[4] A. Puce , V. Ferraresi , R. Biagini , S. Soddu , and R. Loria , “Three‐Dimensional Preclinical Models for Osteosarcoma: Advances and Translational Prospects,” Biomedicine & Pharmacotherapy 191 (2025): 118471, 10.1016/j.biopha.2025.118471. 40848340

[5] A. J. Saraf , J. M. Fenger , and R. D. O. Roberts , “Osteosarcoma: Accelerating Progress Makes for a Hopeful Future,” Frontiers in Oncology 8, no. 4 (2018): 4, 10.3389/fonc.2018.00004. 29435436 PMC5790793

[6] T. Chow , I. Wutami , E. Lucarelli , P. F. Choong , S. Duchi , and C. Di Bella , “Creating In Vitro Three‐Dimensional Tumor Models: A Guide for the Biofabrication of a Primary Osteosarcoma Model,” Tissue Engineering Part B: Reviews 27 (2021): 514–529, 10.1089/ten.TEB.2020.0254. 33138724

[7] M. Hay , D. W. Thomas , J. L. Craighead , C. Economides , and J. Rosenthal , “Clinical Development Success Rates for Investigational Drugs,” Nature Biotechnology 32 (2014): 40–51, 10.1038/nbt.2786. 24406927

[8] P. G. Mthethwa , L. C. Marais , V. Ramsuran , and C. M. Aldous , “A Systematic Review of the Heterogenous Gene Expression Patterns Associated With Multidrug Chemoresistance in Conventional Osteosarcoma,” Genes 14 (2023): 832, 10.3390/genes14040832. 37107591 PMC10137822

[9] H. C. Beird , S. S. Bielack , A. M. Flanagan , et al., “Osteosarcoma,” Nature Reviews Disease Primers 8 (2022): 77, 10.1038/s41572-022-00409-y. 36481668

[10] J. de Azevedo , T. Fernandes , J. Fernandes , et al., “Biology and Pathogenesis of Human Osteosarcoma (Review),” Oncology Letters 19 (2020): 1099–1116, 10.3892/ol.2019.11229. 31966039 PMC6955653

[11] L. Ottaviano , K.‐L. Schaefer , M. Gajewski , et al., “Molecular Characterization of Commonly Used Cell Lines for Bone Tumor Research: A Trans‐European EuroBoNet Effort,” Genes, Chromosomes and Cancer 49 (2010): 40–51, 10.1002/gcc.20717. 19787792

[12] T. M. Fan , R. D. Roberts , and M. M. Lizardo , “Understanding and Modeling Metastasis Biology to Improve Therapeutic Strategies for Combating Osteosarcoma Progression,” Frontiers in Oncology 10 (2020): 13, 10.3389/fonc.2020.00013. 32082995 PMC7006476

[13] M. Cortini , S. Avnet , and N. Baldini , “Mesenchymal Stroma: Role in Osteosarcoma Progression,” Cancer Letters 405 (2017): 90–99, 10.1016/j.canlet.2017.07.024. 28774797

[14] X. S. Cai , Y. W. Jia , J. Mei , and R. Y. Tang , “Tumor Blood Vessels Formation in Osteosarcoma: Vasculogenesis Mimicry,” Chinese Medical Journal 117 (2004): 94–98. 14733782

[15] A. B. Mohseny , I. Machado , Y. Cai , et al., “Functional Characterization of Osteosarcoma Cell Lines Provides Representative Models to Study the Human Disease,” Laboratory Investigation 91 (2011): 1195–1205, 10.1038/labinvest.2011.72. 21519327

[16] B. Clarke , “Normal Bone Anatomy and Physiology,” Clinical Journal of the American Society of Nephrology 3 (2008): S131–S139, 10.2215/CJN.04151206. 18988698 PMC3152283

[17] K. M. Park , D. Lewis , and S. Gerecht , “Bioinspired Hydrogels to Engineer Cancer Microenvironments,” Annual Review of Biomedical Engineering 19 (2017): 109–133, 10.1146/annurev-bioeng-071516-044619. PMC5784262 28633560

[18] S. W. Sawyer and M. E. Oest , “Behavior of Encapsulated Saos‐2 Cells Within Gelatin Methacrylate Hydrogels,” Journal of Tissue Science & Engineering 7 (2016): 2, 10.4172/2157-7552.1000173.

[19] Y. Fang and R. M. Eglen , “Three‐Dimensional Cell Cultures in Drug Discovery and Development,” SLAS Discovery 22 (2017): 456–472, 10.1177/1087057117696795. 28520521 PMC5448717

[20] W. Jiao , J. Shan , X. Gong , et al., “GelMA Hydrogel: A Game‐Changer in 3D Tumor Modeling,” Materials Today Chemistry 38 (2024): 102111, 10.1016/j.mtchem.2024.102111.

[21] I. Pepelanova , K. Kruppa , T. Scheper , and A. Lavrentieva , “Gelatin‐Methacryloyl (GelMA) Hydrogels With Defined Degree of Functionalization as a Versatile Toolkit for 3D Cell Culture and Extrusion Bioprinting,” Bioengineering 5 (2018): 55, 10.3390/bioengineering5030055. 30022000 PMC6165498

[22] C. D. O'Connell , B. Zhang , C. Onofrillo , et al., “Tailoring the Mechanical Properties of Gelatin Methacryloyl Hydrogels Through Manipulation of the Photocrosslinking Conditions,” Soft Matter 14 (2018): 2142–2151, 10.1039/c7sm02187a. 29488996

[23] L. E. Jansen , N. P. Birch , J. D. Schiffman , A. J. Crosby , and S. R. Peyton , “Mechanics of Intact Bone Marrow,” Journal of the Mechanical Behavior of Biomedical Materials 50 (2015): 299–307, 10.1016/j.jmbbm.2015.06.023. 26189198 PMC4554886

[24] K. W. Ng , D. T. Leong , and D. W. Hutmacher , “The Challenge to Measure Cell Proliferation in Two and Three Dimensions,” Tissue Engineering 11 (2005): 182–191, 10.1089/ten.2005.11.182. 15738673

[25] W. H. Abuwatfa , W. G. Pitt , and G. A. Husseini , “Scaffold‐Based 3D Cell Culture Models in Cancer Research,” Journal of Biomedical Science 31 (2024): 7, 10.1186/s12929-024-00994-y. 38221607 PMC10789053

[26] R. Hoffman , “Green Fluorescent Protein Imaging of Tumour Growth, Metastasis, and Angiogenesis in Mouse Models,” Lancet Oncology 3 (2002): 546–556, 10.1016/s1470-2045(02)00848-3. 12217792

[27] T. F. Pereira , G. Levin , C. DeOcesano‐Pereira , et al., “Fluorescence‐Based Method Is More Accurate Than Counting‐Based Methods for Plotting Growth Curves of Adherent Cells,” BMC Research Notes 13 (2020): 57, 10.1186/s13104-020-4914-8. 32019595 PMC7001368

[28] J. Zolnierowicz , M. Ambrozek‐Latecka , J. Kawiak , D. Wasilewska , and G. Hoser , “Monitoring Cell Proliferation In Vitro With Different Cellular Fluorescent Dyes,” Folia Histochemica et Cytobiologica 51 (2013): 193–200, 10.5603/FHC.2013.0027. 24203624

[29] K. Doi , J. Hargitai , J. Kong , et al., “Lentiviral Transduction of Green Fluorescent Protein in Retinal Epithelium: Evidence of Rejection,” Vision Research 42 (2002): 551–558, 10.1016/s0042-6989(01)00237-1. 11853772

[30] Y. Su , X. Luo , B.‐C. He , et al., “Establishment and Characterization of a New Highly Metastatic Human Osteosarcoma Cell Line,” Clinical & Experimental Metastasis 26 (2009): 599–610, 10.1007/s10585-009-9259-6. 19363654

[31] Y. Tome , S. Yano , N. Sugimoto , et al., “Use of α v Integrin Linked to GFP to Image Molecular Dynamics in Trafficking Cancer‐Cell Emboli,” Journal of Cellular Biochemistry 118 (2017): 26–30, 10.1002/jcb.25603. 27191371

[32] C. F. Monteiro , I. A. Deus , I. B. Silva , I. F. Duarte , C. A. Custódio , and J. F. Mano , “Tumor‐on‐a‐Chip Model Incorporating Human‐Based Hydrogels for Easy Assessment of Metastatic Tumor Inter‐Heterogeneity,” Advanced Functional Materials 34 (2024): 2315940, 10.1002/adfm.202315940.

[33] M. K. Kauffman , M. E. Kauffman , H. Zhu , Z. Jia , and Y. R. Li , “Fluorescence‐Based Assays for Measuring Doxorubicin in Biological Systems,” Reactive Oxygen Species 2 (2016): 432–439, 10.20455/ros.2016.873. 29707647 PMC5921830

[34] M. Serra , M. Pasello , M. C. Manara , et al., “May P‐Glycoprotein Status be Used to Stratify High‐Grade Osteosarcoma Patients? Results From the Italian/Scandinavian Sarcoma Group 1 Treatment Protocol,” International Journal of Oncology 29 (2006): 1459–1468. 17088985

[35] M. Serra , D. Maurici , K. Scotlandi , et al., “Relationship Between P‐Glycoprotein Expression and p53 Status in High‐Grade Osteosarcoma,” International Journal of Oncology 14 (1999): 301–307, 10.3892/ijo.14.2.301. 9917506

[36] E. Palmerini , M. R. Sapienza , S. A. Pileri , et al., “Tumor Immune Microenvironment–Associated Prognostic and Mifamurtide‐Response Gene Signatures for Localized Osteosarcoma: A Correlative Study of the ISG/OS‐2 Trial,” Clinical Cancer Research 31 (2025): 3932–3943, 10.1158/1078-0432.CCR-25-0649. 40711479

[37] R. M. Hoffman , “Application of GFP Imaging in Cancer,” Laboratory Investigation 95 (2015): 432–452, 10.1038/labinvest.2014.154. 25686095 PMC4383682

[38] M. R. Soboleski , J. Oaks , and W. P. Halford , “Green Fluorescent Protein Is a Quantitative Reporter of Gene Expression in Individual Eukaryotic Cells,” Federation of American Societies for the FASEB Journal 19 (2005): 1–20, 10.1096/fj.04-3180fje. PMC1242169 15640280

[39] S. Lenna , C. Bellotti , S. Duchi , et al., “Mesenchymal Stromal Cells Mediated Delivery of Photoactive Nanoparticles Inhibits Osteosarcoma Growth In Vitro and in a Murine In Vivo Ectopic Model,” Journal of Experimental & Clinical Cancer Research 39 (2020): 40, 10.1186/s13046-020-01548-4. 32087737 PMC7036176

[40] S. Dini , B. J. Binder , S. C. Fischer , et al., “Identifying the Necrotic Zone Boundary in Tumour Spheroids With Pair‐Correlation Functions,” Journal of the Royal Society Interface 13 (2016): 20160649, 10.1098/rsif.2016.0649. 27733696 PMC5095222

[41] M. Kansara , M. W. Teng , M. J. Smyth , and D. M. Thomas , “Translational Biology of Osteosarcoma,” Nature Reviews Cancer 14 (2014): 722–735, 10.1038/nrc3838. 25319867

[42] J. Ritter and S. S. O. Bielack , “Osteosarcoma,” Annals of Oncology 21, (2010): vii320–vii325, 10.1093/annonc/mdq276. 20943636

[43] H. V. Wadell , “Volume, Shape, and Roundness of Quartz Particles,” Journal of Geology 43 (1935): 250–280, 10.1086/624298.

[44] S. J. Kuerbitz and M. B. O. Henderson , “Osteosarcoma: A Review With Emphasis on Pathogenesis and Chemoresistance,” Medical Research Archives 8 (2020): 2170, 10.18103/mra.v8i7.2170.

[45] M. Basotra , S. K. Singh , and M. Gulati , “Development and Validation of a Simple and Sensitive Spectrometric Method for Estimation of Cisplatin Hydrochloride in Tablet Dosage Forms: Application to Dissolution Studies,” ISRN Analytical Chemistry 2013 (2013): 1–8, 10.1155/2013/936254.

[46] P. Rajkumar , “Cisplatin Concentrations in Long and Short Duration Infusion: Implications for the Optimal Time of Radiation Delivery,” Journal of Clinical and Diagnostic Research 10 (2016): XC01–XC04, 10.7860/JCDR/2016/18181.8126. PMC5020194 27630935

[47] B. Peng , M. W. English , A. V. Boddy , et al., “Cisplatin Pharmacokinetics in Children With Cancer,” European Journal of Cancer 33 (1997): 1823–1828, 10.1016/s0959-8049(97)00341-9. 9470840

[48] A. R. Jørgensen , M. Bue , P. Hanberg , et al., “Doxorubicin Concentrations in Bone Tumour‐Relevant Tissues After Bolus and Continuous Infusion: A Randomized Porcine Microdialysis Study,” Cancer Chemotherapy and Pharmacology 93 (2024): 555–564, 10.1007/s00280-023-04637-1. 38332155 PMC11130026

[49] M. A. G. Barbosa , C. P. R. Xavier , R. F. Pereira , V. Petrikaite , and M. H. Vasconcelos , “3D Cell Culture Models as Recapitulators of the Tumor Microenvironment for the Screening of Anti‐Cancer Drugs,” Cancers 14 (2021): 190, 10.3390/cancers14010190. 35008353 PMC8749977

[50] R. N. Ghosh , J. Thomas , V. B R , et al., “An Insight Into Synthesis, Properties and Applications of Gelatin Methacryloyl Hydrogel for 3D Bioprinting,” Materials Advances 4 (2023): 5496–5529, 10.1039/d3ma00715d.

[51] Y. Lin , K. Yuan , Y. Yang , et al., “Osteosarocma Progression in Biomimetic Matrix With Different Stiffness: Insights From a Three‐Dimensional Printed Gelatin Methacrylamide Hydrogel,” International Journal of Biological Macromolecules 252 (2023): 126391, 10.1016/j.ijbiomac.2023.126391. 37595702

[52] K. Menshikh , I. Banicevic , B. Obradovic , and L. Rimondini , “Biomechanical Aspects in Bone Tumor Engineering,” Tissue Engineering Part B: Reviews 30 (2024): 217–229, 10.1089/ten.TEB.2023.0106. 37830183 PMC11001506

[53] J. S. Uzarski , M. D. DiVito , J. A. Wertheim , and W. M. Miller , “Essential Design Considerations for the Resazurin Reduction Assay to Noninvasively Quantify Cell Expansion Within Perfused Extracellular Matrix Scaffolds,” Biomaterials 129 (2017): 163–175, 10.1016/j.biomaterials.2017.02.015. 28343003 PMC5765551

[54] J. L. Dahlin , Assay Guidance Manual, ed. S. Markossian , A. Grossman , and H. Baskir , (Eli Lilly & Company and the National Center for Advancing Translational Sciences, 2004). 22553861

[55] K. E. Gascoigne and S. S. Taylor , “How Do Anti‐Mitotic Drugs Kill Cancer Cells?,” Journal of Cell Science 122 (2009): 2579–2585, 10.1242/jcs.039719. 19625502

[56] M. A. Jordan and L. Wilson , “Microtubules as a Target for Anticancer Drugs,” Nature Reviews Cancer 4 (2004): 253–265, 10.1038/nrc1317. 15057285

[57] J. He , Z. Qiu , J. Fan , X. Xie , Q. Sheng , and X. Sui , “Drug Tolerant Persister Cell Plasticity in Cancer: A Revolutionary Strategy for More Effective Anticancer Therapies,” Signal Transduction and Targeted Therapy 9 (2024): 209, 10.1038/s41392-024-01891-4. 39138145 PMC11322379

[58] E. Delage , T. Guilbert , and F. Yates , “Successful 3D Imaging of Cleared Biological Samples With Light Sheet Fluorescence Microscopy,” Journal of Cell Biology 222 (2023): 202307143, 10.1083/jcb.202307143. PMC10583220 37847528

[59] M. Pavlou , M. Shah , P. Gikas , T. Briggs , S. J. Roberts , and U. Cheema , “Osteomimetic Matrix Components Alter Cell Migration and Drug Response in a 3D Tumour‐Engineered Osteosarcoma Model,” Acta Biomaterialia 96 (2019): 247–257, 10.1016/j.actbio.2019.07.011. 31302294

[60] M. V. Monteiro , V. M. Gaspar , L. P. Ferreira , and J. F. Mano , “Hydrogel 3D In Vitro Tumor Models for Screening Cell Aggregation Mediated Drug Response,” Biomaterials Science 8 (2020): 1855–1864, 10.1039/c9bm02075f. 32091033

[61] D. A. Muller and U. Silvan , “On the Biomechanical Properties of Osteosarcoma Cells and Their Environment,” International Journal of Developmental Biology 63 (2019): 1–8, 10.1387/ijdb.190019us. 30919911

[62] R. Song , K. Tian , W. Wang , and L. Wang , “P53 Suppresses Cell Proliferation, Metastasis, and Angiogenesis of Osteosarcoma Through Inhibition of the PI3K/AKT/mTOR Pathway,” International Journal of Surgery 20 (2015): 80–87, 10.1016/j.ijsu.2015.04.050. 25936826

[63] M. Zenmyo , S. Komiya , T. Hamada , et al., “Transcriptional Activation of p21 by Vitamin D3 or Vitamin K2 Leads to Differentiation of p53‐deficient MG‐63 Osteosarcoma Cells,” Human Pathology 32 (2001): 410–416, 10.1053/hupa.2001.23524. 11331958

[64] Y. Xie , A. Mustafa , A. Yerzhan , et al., “Nuclear Matrix Metalloproteinases: Functions Resemble the Evolution From the Intracellular to the Extracellular Compartment,” Cell Death Discovery 3 (2017): 17036, 10.1038/cddiscovery.2017.36. 28811933 PMC5554797

[65] J.‐Y. Feng , X.‐F. Wei , L. Chen , et al., “SOCS1 Depletion Drives Osteosarcoma Stemness and Chemoresistance by Suppressing ACTN4 Degradation,” Acta Pharmacologica Sinica 47 (2026): 255–271, 10.1038/s41401-025-01650-3. 40890444 PMC12764541

[66] J. Liu , Y. Xu , T. Xu , et al., “MUC1 Promotes Cancer Stemness and Predicts Poor Prognosis in Osteosarcoma,” Pathology—Research and Practice 242 (2023): 154329, 10.1016/j.prp.2023.154329. 36680928

[67] J. W. Martin , M. Zielenska , G. S. Stein , A. J. van Wijnen , and J. A. Squire , “The Role of RUNX2 in Osteosarcoma Oncogenesis,” Sarcoma 2011 (2011): 1–13, 10.1155/2011/282745. PMC3005824 21197465

[68] Y. Li , C. Ge , J. P. Long , et al., “Biomechanical Stimulation of Osteoblast Gene Expression Requires Phosphorylation of the RUNX2 Transcription Factor,” Journal of Bone and Mineral Research 27 (2012): 1263–1274, 10.1002/jbmr.1574. 22337141 PMC3532028

[69] J. Shi and P. K. Wei , “Interleukin‐8: A Potent Promoter of Angiogenesis in Gastric Cancer,” Oncology Letters 11 (2016): 1043–1050, 10.3892/ol.2015.4035. 26893688 PMC4734231

[70] R. Tatsuno , J. Ichikawa , Y. Komohara , et al., “Pivotal Role of IL‐8 Derived From the Interaction Between Osteosarcoma and Tumor‐Associated Macrophages in Osteosarcoma Growth and Metastasis via the FAK Pathway,” Cell Death & Disease 15 (2024): 108, 10.1038/s41419-024-06487-y. 38302407 PMC10834992

[71] A. DiNatale , M. S. Castelli , B. Nash , O. Meucci , and A. Fatatis , “Regulation of Tumor and Metastasis Initiation by Chemokine Receptors,” Journal of Cancer 13 (2022): 3160–3176, 10.7150/jca.72331. 36118530 PMC9475358

[72] E. Torreggiani , L. Roncuzzi , F. Perut , N. Zini , and N. Baldini , “Multimodal Transfer of MDR by Exosomes in Human Osteosarcoma,” International Journal of Oncology 49 (2016): 189–196, 10.3892/ijo.2016.3509. 27176642

[73] Z. Zhou , L. Wan , Y. Han , et al., “ABCB1‐Overexpressing MG63/DOX Cell Xenograft Model: Maintain the MDR Phenotype In Vivo,” Pharmaceutical Biology 51 (2013): 968–973, 10.3109/13880209.2013.772640. 23735077

[74] J. C. Fontoura , C. Viezzer , F. G. dos Santos , et al., “Comparison of 2D and 3D Cell Culture Models for Cell Growth, Gene Expression and Drug Resistance,” Materials Science and Engineering: C 107 (2020): 110264, 10.1016/j.msec.2019.110264. 31761183

[75] P. H. Tan , S. S. Chia , S. L. Toh , J. C. Goh , and S. S. Nathan , “Three‐Dimensional Spatial Configuration of Tumour Cells Confers Resistance to Chemotherapy Independent of Drug Delivery,” Journal of Tissue Engineering and Regenerative Medicine 10 (2016): 637–646, 10.1002/term.1800. 24668783

[76] V. Sandhu , D. Bakkalci , S. Wei , and U. Cheema , “Enhanced Biomimetics of Three‐Dimensional Osteosarcoma Models: A Scoping Review,” Cancers 16 (2023): 164, 10.3390/cancers16010164. 38201591 PMC10778420

[77] C.‐J. Ho , H.‐J. Ko , T.‐S. Liao , et al., “Severe Cellular Stress Activates Apoptosis Independently of p53 in Osteosarcoma,” Cell Death Discovery 7 (2021): 275, 10.1038/s41420-021-00658-y. 34608124 PMC8490387

[78] C. R. Dass and P. F. Choong , “GFP Expression Alters Osteosarcoma Cell Biology,” DNA and Cell Biology 26 (2007): 599–601, 10.1089/dna.2006.0531. 17688411

[79] T. Chow , W. Humble , E. Lucarelli , et al., “Feasibility and Barriers to Rapid Establishment of Patient‐Derived Primary Osteosarcoma Cell Lines in Clinical Management,” Iscience 27 (2024): 110251, 10.1016/j.isci.2024.110251. 39286504 PMC11403063

[80] E. C. González Díaz , A. G. Lee , L. C. Sayles , C. Feria , E. A. Sweet‐Cordero , and F. Yang , “A 3D Osteosarcoma Model With Bone‐Mimicking Cues Reveals a Critical Role of Bone Mineral and Informs Drug Discovery,” Advanced Healthcare Materials 11 (2022): 2200768, 10.1002/adhm.202200768. PMC10162498 35767377

[81] C. Salameh , F. Salviat , E. Bessot , et al., “Origin of Transparency in Scattering Biomimetic Collagen Materials,” Proceedings of the National Academy of Sciences of the United States of America 117 (2020): 11947–11953, 10.1073/pnas.2001178117. 32424103 PMC7275709

[82] G. Bassi , M. Saqawa , L. Apolloni , et al., “Deciphering the Interaction Between Osteosarcoma and Mesenchymal Stem Cells in a 3D Bone‐Mimetic Co‐Culture Model,” Biomedicine & Pharmacotherapy 195 (2026): 118956, 10.1016/j.biopha.2025.118956. 41496360

[83] Y. Zhao , X. Chen , S. Bai , et al., “Fabrication of Gelatin Methacrylate/Nanohydroxyapatite Microgel Arrays for Periodontal Tissue Regeneration,” International Journal of Nanomedicine 11 (2016): 4707–4718, 10.2147/IJN.S111701. 27695327 PMC5028089

[84] Y. Zhu , X. Yu , H. Liu , et al., “Strategies of Functionalized GelMA‐Based Bioinks for Bone Regeneration: Recent Advances and Future Perspectives,” Bioactive Materials 38 (2024): 346–373, 10.1016/j.bioactmat.2024.04.032. 38764449 PMC11101688

[85] E. Martella , C. Ferroni , A. Guerrini , et al., “Functionalized Keratin as Nanotechnology‐Based Drug Delivery System for the Pharmacological Treatment of Osteosarcoma,” International Journal of Molecular Sciences 19 (2018): 3670, 10.3390/ijms19113670. 30463350 PMC6274803

[86] H. Wadell , “Sphericity and Roundness of Rock Particles,” Journal of Geology 41 (1933): 310–331, 10.1086/624040.

[87] A. A. Dussault and M. Pouliot , “Rapid and Simple Comparison of Messenger RNA Levels Using Real‐Time PCR,” Biological Procedures Online 8 (2006): 1–10, 10.1251/bpo114. 16446781 PMC1352391

