# Supplementary material for: FOS3D: A Fluorescence‐Enabled Toolkit for Characterizing a Three‐dimensional Osteosarcoma Model
Source: Adv Sci (Weinh). 2026 Jun 11:e76031. Online ahead of print. doi: 10.1002/advs.76031 (PMC13336488; doi:10.1002/advs.76031)
Supplement: Supplementary file 1 — Supporting File 1: advs76031‐sup‐0001‐SuppMat.docx [file ADVS-9999-e76031-s002.docx]

# **Supplementary Data**

**FOS3D: A Fluorescence-Enalbed Toolkit for Characterising a Three-Dimensional Osteosarcoma Model**

William Humble^1,2^, Wiktor Zywicki^3^, Enrico Lucarelli^4^, Ania Naila Guerrieri^4^, Francesca Taraballi^5,6^, Gianluca Cidonio^7,8^, Claudia Di Bella^1,2,9^, Carmine Onofrillo^1,2^, Andrea J. O’Connor ^1,3^, Serena Duchi ^1,2*^

Affiliations:

^1^Aikenhead Centre for Medical Discovery (ACMD), St Vincent's Hospital Melbourne, Fitzroy, VIC, Australia.

^2^Department of Surgery, The University of Melbourne, St Vincent's Hospital Melbourne, Fitzroy, VIC, Australia.

^3^Department of Biomedical Engineering, The Graeme Clark Institute, The University of Melbourne, Parkville, VIC, Australia.

^4^Osteoncology, Bone and Soft Tissue Sarcomas and Innovative Therapies Unit, IRCCS Istituto Ortopedico Rizzoli, Via di Barbiano 1/10, 40136 Bologna, Italy.

^5^Center for Musculoskeletal Regeneration, Houston Methodist Research Institute, Houston, TX 77030, United States of America.

^6^Orthopaedics and Sports Medicine, Houston Methodist Hospital, Houston, TX 77030, United States of America.

^7^Department of Mechanical and Aerospace Engineering, University of Rome 'La Sapienza', 00184 Rome, Italy.

^8^Center for Life Nano- & Neuro- Science-CLN2S, Italian Institute of Technology (IIT), 00161 Rome, Italy.

^9^Department of Orthopaedics, Sarcoma Unit, St Vincent's Hospital Melbourne, Fitzroy, VIC 3065, Australia.

Keywords: Osteosarcoma, GelMa, Fluorescence, In Vitro Models, Tissue Engineering, Chemotherapeutics.

*Corresponding author: Serena Duchi, sduchi@unimelb.edu.au


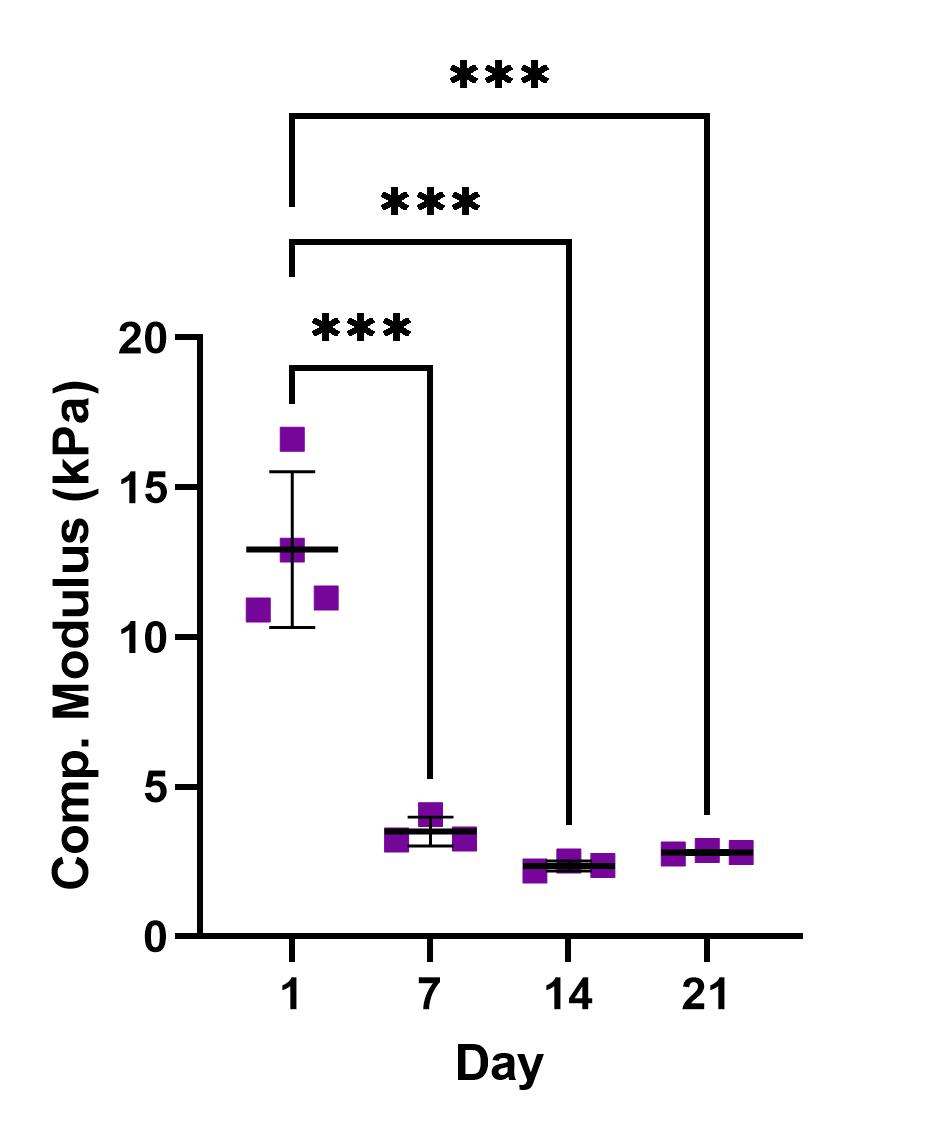


**Figure S1.** Compressive modulus of 35µl (casted in 3.3 mm^3^ molds) hydrogels post-swelling determined within the strain range of 10 – 15% and at several timepoints, using a BOSE Electroforce 3200 compressor. Hydrogels are seeded with 70,000 MG-63-GFP cells at time of biofabrication. n = 4 technical replicates per timepoint within the same hydrogel batch.

**Supplemental Video 1.** Video depicts three-dimensional animation of a Day 1, 35µL GelMA hydrogel containing fluorescence expressing MG-63-GFP cells rotating in space. After a brief period, the green signal from the fluorescent cells is converted to Spots based on the average cell circumference. These Spots allow visual representation of cells and statistical analysis. Acquisition: 4X, GFP (488nm) laser, scalebar represents 300µm. Images were acquired with UltraMicroscope Blaze™ light sheet microscope (Miltenyi Biotec, Germany). Image processing conducted using Imaris10 (Oxford Instruments, UK).

**Supplemental Video 2.** Video depicts three-dimensional animation of a Day 7, 35µL GelMA hydrogel containing fluorescence expressing MG-63-GFP cells rotating in space. After a brief period, the green signal from the fluorescent cells is converted to Spots based on the average cell circumference. These Spots allow visual representation of cells and statistical analysis. Acquisition: 4X, GFP (488nm) laser, scalebar represents 300µm. Images were acquired with UltraMicroscope Blaze™ light sheet microscope (Miltenyi Biotec, Germany). Image processing conducted using Imaris10 (Oxford Instruments, UK).

**Supplemental Video 3.** Video depicts three-dimensional animation at the surface region of a Day 1, 35µL GelMA hydrogel containing fluorescence expressing MG-63-GFP cells. After a brief period, the green signal from the fluorescent cells is converted to spots using the Spot function in Imaris10 (Oxford Instruments, UK), estimating the centre of each cell. After another brief period the signal from cytosolic expression of green fluorescent protein is converted to an object surface using the Surface function in Imaris10 (Oxford Instruments, UK). This allows aggregation and spreading of cells to be quantified using in-built statistical analysis testing for sphericity. Acquisition: 12X, GFP (488nm) laser, z-step = 20µm, stack height = 500µm, scalebar represents 100µm. Images were acquired with UltraMicroscope Blaze™ light sheet microscope (Miltenyi Biotec, Germany).

**Supplemental Video 4.** Video depicts three-dimensional animation at the surface region of a Day 7, 35µL GelMA hydrogel containing fluorescence expressing MG-63-GFP cells. After a brief period, the green signal from the fluorescent cells is converted to spots using the Spot function in Imaris10 (Oxford Instruments, UK), estimating the centre of each cell. After another brief period the signal from cytosolic expression of green fluorescent protein is converted to an object surface using the Surface function in Imaris10 (Oxford Instruments, UK). This allows aggregation and spreading of cells to be quantified using in-built statistical analysis testing for sphericity. Acquisition: 12X, GFP (488nm) laser, z-step = 20µm, stack height = 500µm, scalebar represents 100µm. Images were acquired with UltraMicroscope Blaze™ light sheet microscope (Miltenyi Biotec, Germany).

**
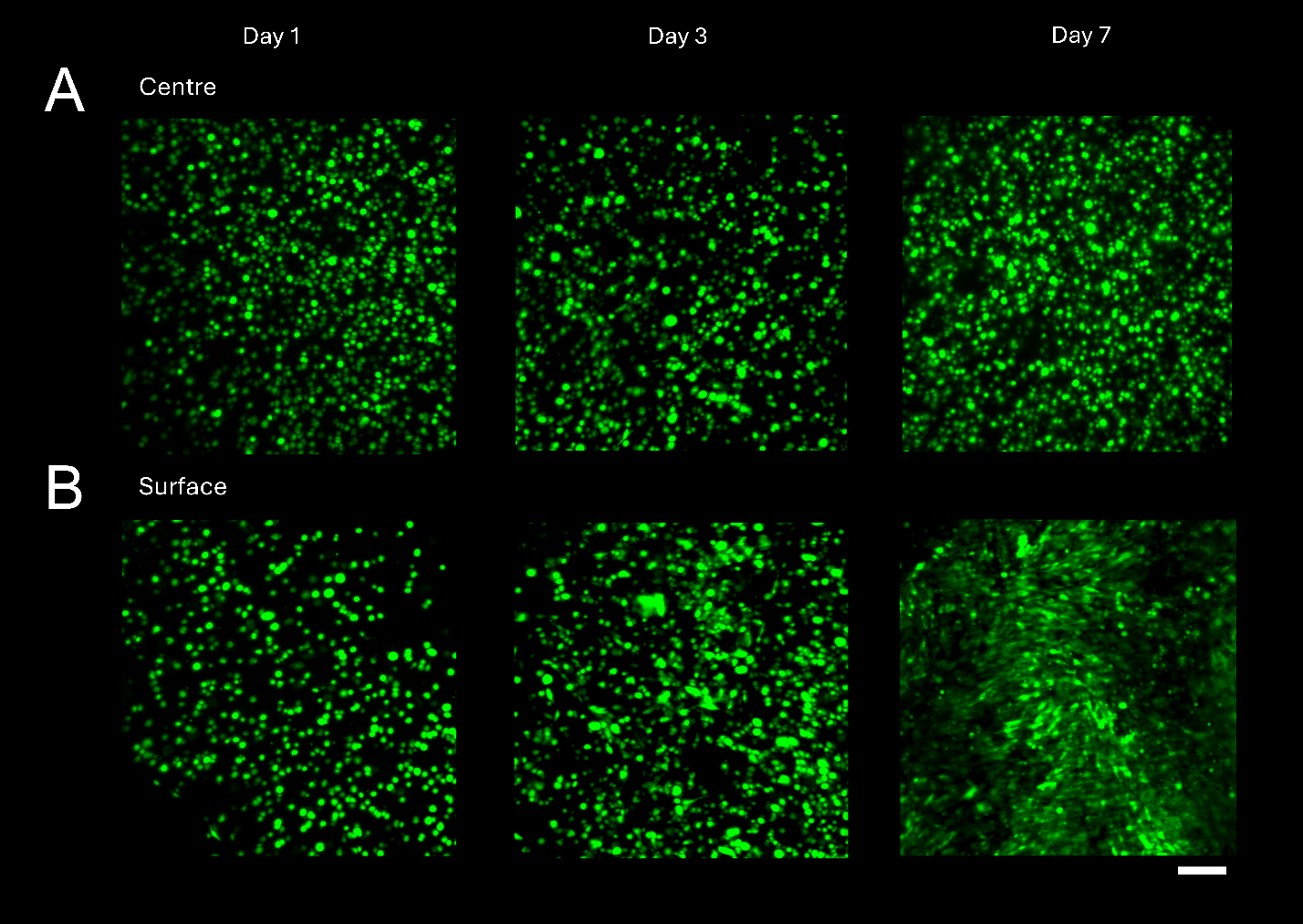
**

**Figure S2.** Representative images capturing fluorescence expressing MG-63-GFP cells (green) in 35µL GelMA hydrogel over seven days culture from the Centre (1mm depth) and Surface regions. Acquisition: 12X, GFP (488nm) laser, z-step = 20µm, stack height = 500µm, scalebar represents 150µm. Images were acquired with UltraMicroscope Blaze™ light sheet microscope (Miltenyi Biotec, Germany).


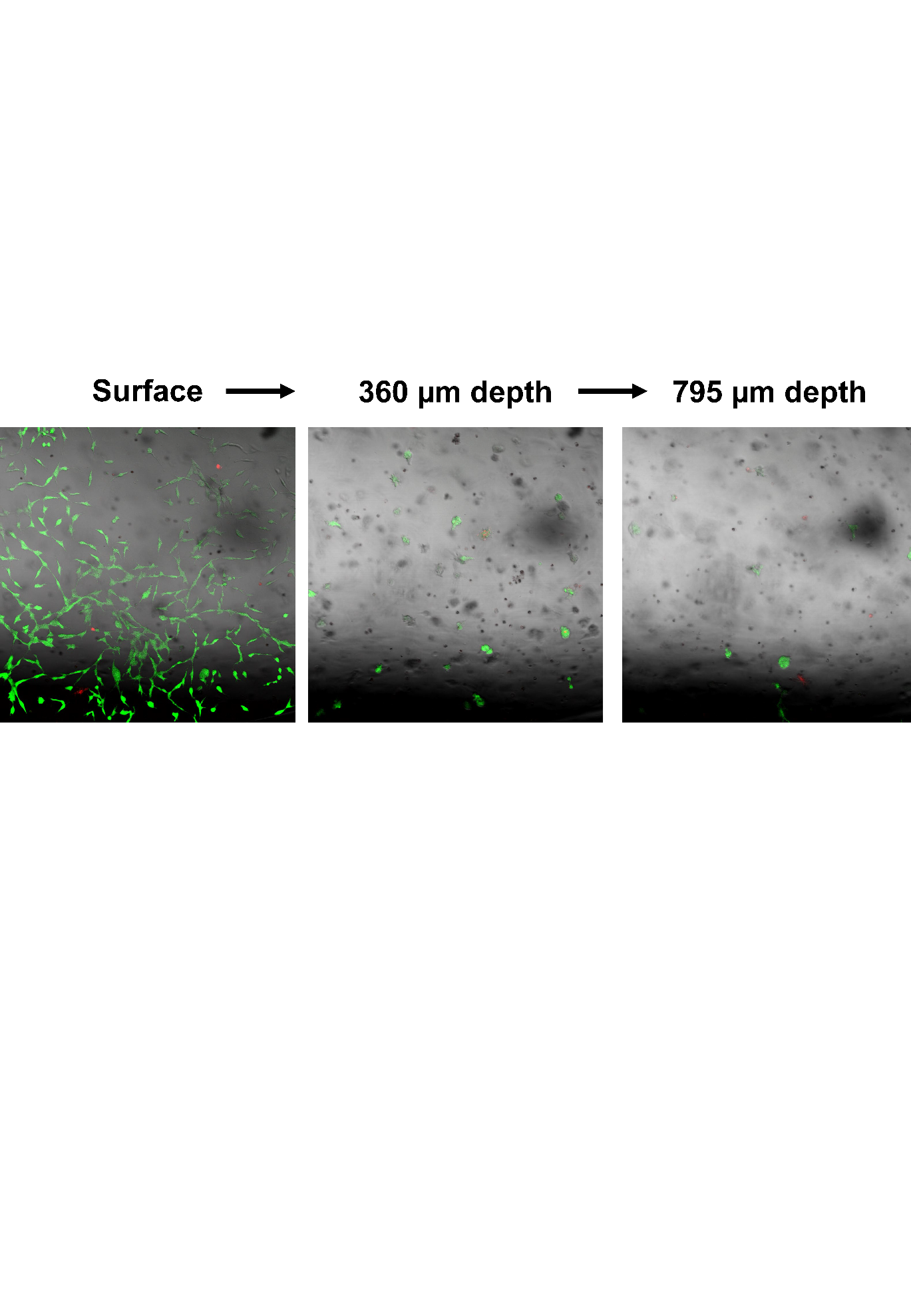


**Figure S3.** Confocal composite images panning through 0.8mm depth from surface to centre of 35µL GelMA hydrogels laden with MG-63-GFP osteosarcoma cells after seven days culture. Live cells are stained with calcein acetoxymethyl ester (green), dead cells with ethidium homodimer (red). Acquisition: 10X, bright-field, GFP, and RFP lasers, Z-stack = 15µm. Composite image created using ImageJ (NIH, USA).

| **TARGET NAME** | **Primers sequences 5’-3’/RefSeq Accession number or Probe assay number** | **Manufacturer** |
| --- | --- | --- |
| β-actin | Fw:AGCCATGCCAATCTCATCTTGTT  Rev:ATCGTCCACCGCAAATGCTTCTA  or  Hs99999903_m1 | International DNA Technologies (IDT), Coralville, USA  Life Technologies |
| TP53 | Fw: TAACAGTTCCTGCATGGGC  Rev:AGGACAGGCACAAACACGC | IDT |
| p21/CDKN1A | Fw:CTGGAGACTCTCAGGGTCGAAA  Rev:GATTAGGGCTTCCTCTTGGAGAA | IDT |
| BAX | NM_004324.3 / PPH00078B | Qiagen |
| PUMA | NM_014417.4 / PPH02204C | Qiagen |
| MMP3 | Fw:AGTCTTCCAATCCTACTGTTGCT Rev:TCCCCGTCACCTCCAATCC | IDT |
| MMP13 | Fw:GACTTCCCAGGAATTGGTGA Rev:TGACGCGAACAATACGGTTA | IDT |
| MMP14 | Fw:GGCTACAGCAATATGGCTACC  Rev:GATGGCCGCTGAGAGTGAC | IDT |
| RUNX-2 | Fw:TGATGACACTGCCACCTCTGA  Rev: GCACCTGCCTGGCTCTTCT | IDT |
| NANOG | Fw:ACCTACCCCAGCCTTTACTC  Rev:GGACTGGATGTTCTGGGTCT | IDT |
| OCT-4 | Fw:TGAGTAGTCCCTTCGCAAGC  Rev:GAGAAGGCGAAATCCGAAGC | IDT |
| SOX-2 | Fw:CGATGCCGACAAGAAAACTT Rev:CAAACTTCCTGCAAAGCTCC | IDT |
| CXCL8 | NM_000584.3 / PPH0568A | Qiagen |
| HIF1α | Fw:TGATGACCAGCAACTTGAGG Rev:CTGGGGCATGGTAAAAGAAA | IDT |
| VEGFA | Fw:CTGGAGTGTGTGCCCACTGA Rev:TCCTATGTGCTGGCCTTGGT | IDT |
| ATR | Hs00992123_m1 | Life Technologies |
| FANCD2 | Hs00276992_m1 | Life Technologies |
| ERCC1 | Hs01012158_m1 | Life Technologies |
| ERCC4(XPF) | Hs00193342_m1 | Life Technologies |
| XRCC3 | Hs00193725_m1 | Life Technologies |
| ABCB1 | Hs00184491_m1 | Life Technologies |

**Table S1.** RT-qPCR probe sequences. Note: some sequences are obscured for copyright reasons.


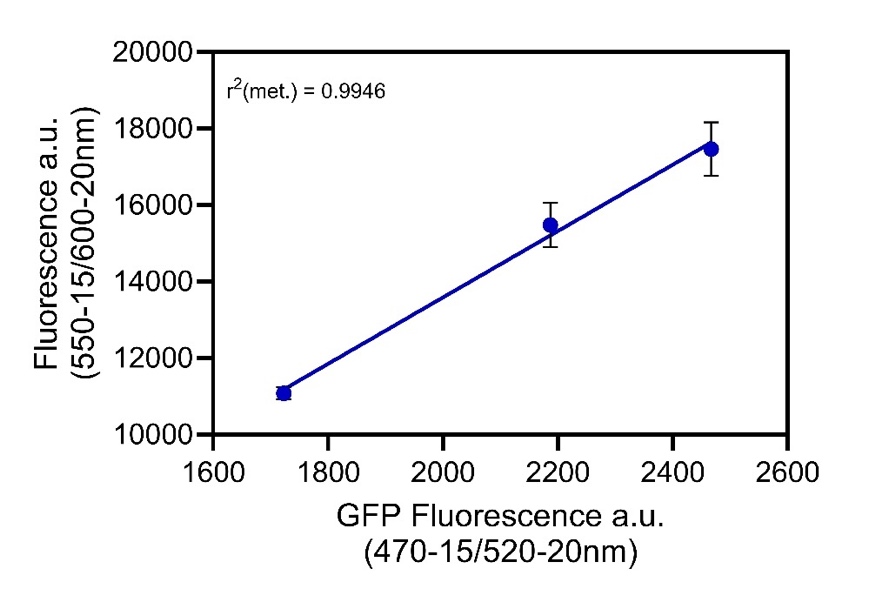


**Figure S4. Saos-2 GFP profiling.** Linear regression analysis (GraphPad Prism 10) reveals correlation between metabolic activity assay (Cell TiterBlue) and well plate reading of endogenous, green fluorescent protein expression (GFP) in Saos-2-GFP osteosarcoma cells over four days. n =3 per timepoint.


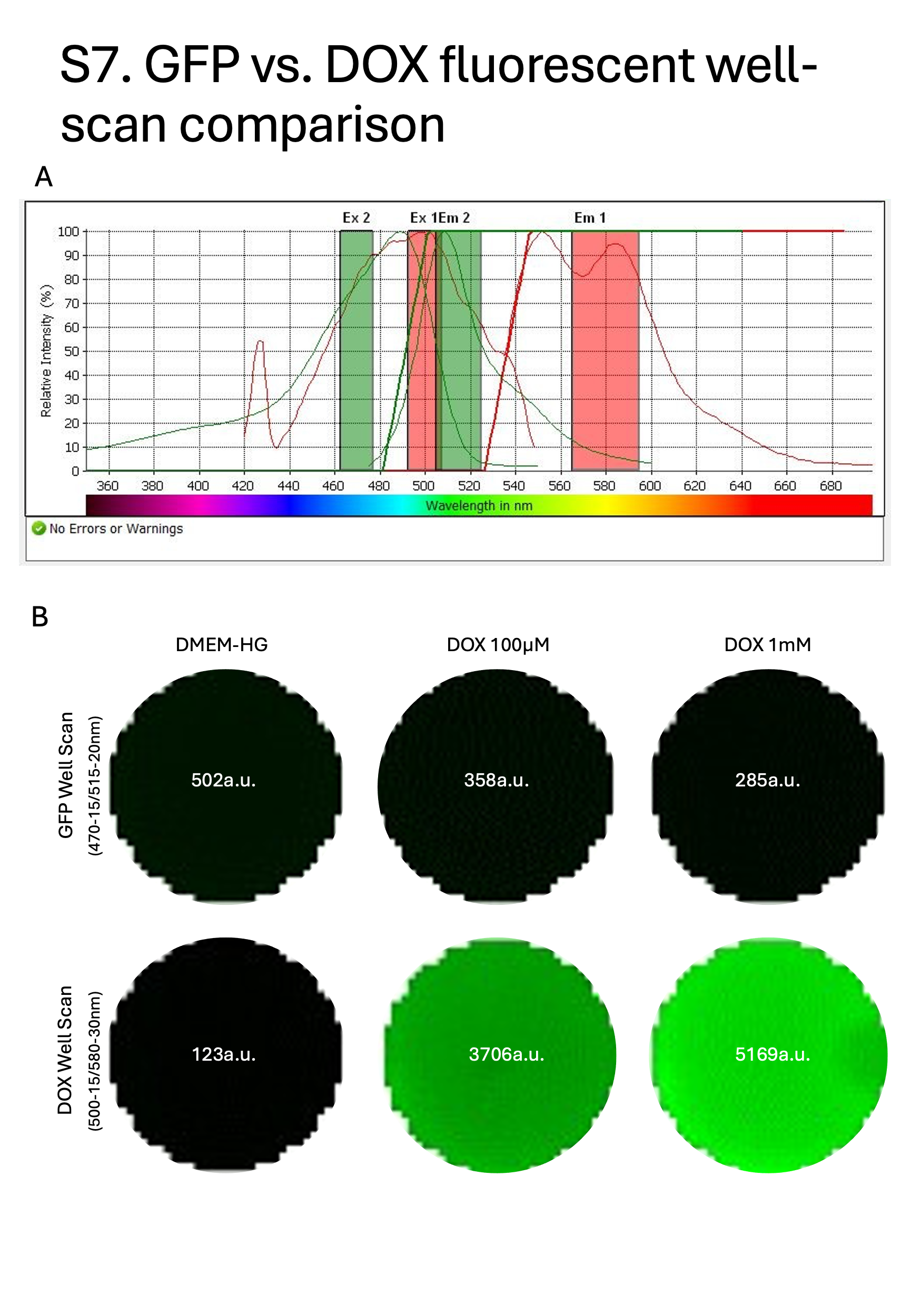


**Figure S5.** (A) Screen-clip of spectral setting for fluorescence intensity reading; GFP depicted in green (dichroic 491.2) and DOX settings depicted in red (dichroic 536.2). (B) Top row shows well scans taken using the GFP excitation/emission spectrum. The additional of high concentrations of DOX diminishes the baseline signal from DMEM-HG. These concentrations greatly exceed those used in *in vitro* drug experiments. Bottom row demonstrates when using the DOX excitation/emission spectrum, the increasing concentrations of DOX are readily observable. The average fluorescence intensity (a.u.) provides quantified data for comparison. GFP = green fluorescent protein, DMEM-HG = dulbecco’s modified eagle medium – high glucose, DOX = doxorubicin, a.u. = arbitrary units.


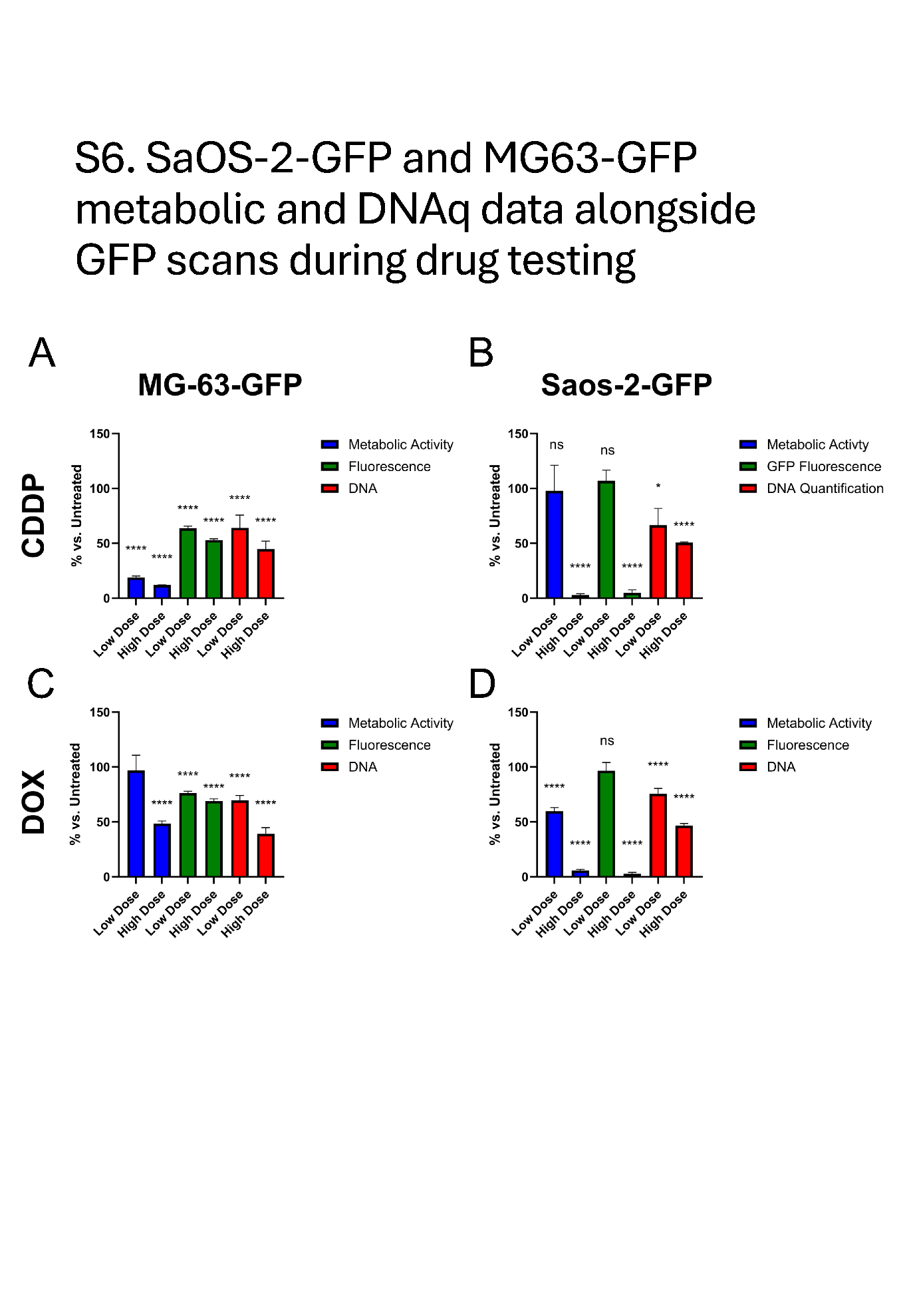


**Figure S6.** (A and C) Cell TiterBlue metabolic assay, and DNA quantification compared to endogenous fluorescence of MG-63-GFP cells treated with either high (120µM) or low (16.3µM) doses of CDDP (A) and comparative assays of MG-63-GFP treated with high (2.97µM) or low (0.11µM) doses of DOX (C). (B and D) Comparative assays of Saos-2-GFP under treatment with respective high (21.6µM) or low (9.43µM) doses of CDDP (B). Comparative assays of Saos-2-GFP treated with high (1.85µM) or low (0.41µM) doses of DOX (D). Treatment groups are displayed as a percentage of the untreated control group for each assay. Statistical analysis comparing treatment to untreated involved one-way ANOVA where **** = p < 0.0001, and * = p < 0.05, ns= non-significant. GFP = green fluorescent protein, CDDP = cisplatin, DOX = doxorubicin.
